# Examining factors related to low performance of predicting remission in participants with major depressive disorder using neuroimaging data and other clinical features

**DOI:** 10.1371/journal.pone.0299625

**Published:** 2024-03-28

**Authors:** Junying Wang, David D. Wu, Christine DeLorenzo, Jie Yang

**Affiliations:** 1 Department of Applied Mathematics and Statistics, Stony Brook University, New York, New York, United states of America; 2 School of Engineering, University of Michigan, Ann Arbor, Michigan, United States of America; 3 Department of Psychiatry and Behavioral Health, Stony Brook University, Stony Brook, New York, United States of America; 4 Department of Biomedical Engineering, Stony Brook University, Stony Brook, New York, United States of America; 5 Department of Family, Population & Preventive Medicine, Stony Brook University, Stony Brook, New York, United States of America; University of Catania Department of Educational Sciences: Universita degli Studi di Catania Dipartimento di Scienze della Formazione, ITALY

## Abstract

Major depressive disorder (MDD), a prevalent mental health issue, affects more than 8% of the US population, and almost 17% in the young group of 18–25 years old. Since Covid-19, its prevalence has become even more significant. However, the remission (being free of depression) rates of first-line antidepressant treatments on MDD are only about 30%. To improve treatment outcomes, researchers have built various predictive models for treatment responses and yet none of them have been adopted in clinical use. One reason is that most predictive models are based on data from subjective questionnaires, which are less reliable. Neuroimaging data are promising objective prognostic factors, but they are expensive to obtain and hence predictive models using neuroimaging data are limited and such studies were usually in small scale (N<100). In this paper, we proposed an advanced machine learning (ML) pipeline for small training dataset with large number of features. We implemented multiple imputation for missing data and repeated K-fold cross validation (CV) to robustly estimate predictive performances. Different feature selection methods and stacking methods using 6 general ML models including random forest, gradient boosting decision tree, XGBoost, penalized logistic regression, support vector machine (SVM), and neural network were examined to evaluate the model performances. All predictive models were compared using model performance metrics such as accuracy, balanced accuracy, area under ROC curve (AUC), sensitivity and specificity. Our proposed ML pipeline was applied to a training dataset and obtained an accuracy and AUC above 0.80. But such high performance failed while applying our ML pipeline using an external validation dataset from the EMBARC study which is a multi-center study. We further examined the possible reasons especially the site heterogeneity issue.

## Introduction

Major depressive disorder (MDD) is diagnosed when an individual experiences five of nine symptoms (either low / depressed mood or anhedonia, and four of the following: feelings of guilt / worthlessness, lack of energy, poor concentration, appetite changes, psychomotor retardation or agitation, sleep disturbances, or suicidal thoughts; American Psychiatric Association, 2013 [1]). It is an extremely common and devastating disease in terms of suffering, mortality and cost to society [2–5]. The challenge of MDD is also growing. Since Covid-19, mental health issues, including major depressive disorder (MDD), have skyrocketed, with a nearly 25% increase in prevalence of anxiety and depression worldwide [6]. MDD is a heterogeneous disorder, with a range of symptoms including cognitive and psychosocial-affective symptoms [7] arising from numerous potential factors: genetic, epigenetic, hypothalamic-pituitary-adrenal (HPA) axis and neurotransmitters [8]. In fact, due to the diagnostic criteria (existence of five of nine symptoms), there are hundreds of ways to meet MDD criteria [9]. This variability in symptoms and etiology likely impacts treatment response, resulting in treatment response heterogeneity [10, 11]. Most patients with MDD do not adequately respond to first line treatment and a large percentage fail multiple interventions [4, 12]. As conventional first line medication treatments require six to eight weeks for full efficacy [12, 13], prediction of antidepressant treatment outcome prior to starting the treatment could prevent ineffective treatment trials and help clinicians to prescribe antidepressant drugs in a more personalized way.

With the hypothesis that integrated multimodal data such as genetic, clinical and demographic data could enable accurate prediction of MDD remission, previous studies combined various clinical, demographic variables and genetic information to predict treatment response using machine learning (ML) methods such as elastic net, random forests, and gradient boosting. Several studies trained their predictive models using the Sequenced Treatment Alternatives to Relieve Depression (STAR*D) dataset, which contained patients’ sociodemographic data, clinical information and a long survey, the psychiatric diagnostic symptom questionnaire, and tested their final models using an external dataset [14–16].

However, none of these predicting models has been adopted in clinical practice due to low performance. This could be because of different sample sizes, effect sizes, publication biases and methodological disparities preventing the strength and directionality of predictors from being accurately accessed [17]. Other reasons could be lacking the selection of clinical or behavioral features as well as demographic variables such as income which was associated with treatment outcome in univariate analysis [12, 15]. In addition, none of these aforementioned predicting models used neuroimaging data. Different from subjective data collected from different questionnaires, neuroimaging data are objective measurements and hence more accurate. It has been recognized that neuroimaging measures may not be powerful for diagnostic classification of MDD, but they may be useful in prediction treatment responses [18–20]. Because of the relatively high expense and technical difficulty to obtain neuroimaging data, studies that collected neuroimaging data are generally in small scale, often with <100 participants.

Our objectives were to develop an advanced ML pipeline for small sample size (<100) with large number of features, where the ML pipeline included data pre-processing, missing value imputation, feature selection, predictive modeling, and model performance evaluation. We used a training dataset from a single-site study and an external validation dataset from a multi-site study to verify the generalization of our ML pipeline from single site to multi-site. We further examined possible reasons for the unsatisfying predictive performance.

## Materials and methods

We used data from two completed clinical trials: APAT study and EMBARC study. We built a predictive modal and internally cross-validated the model using APAT study first. Then we externally validated the model developed in a wholly independent clinical trial, EMBARC study.

### APAT study

APAT study is randomized, placebo-controlled clinical trial titled as Advancing Personalized Antidepressant Treatment Using PET/MRI (ClinicalTrials.gov, NCT02623205), funded by the National Institute of Mental Health (R01MH104512), the DANA Foundation, and the Brain and Behavior Research Foundation. This study is approved by Stony Brook University IRB (#570152). 85 participants with Major Depressive Disorder (MDD) enrolled between 3/20/2015–3/04/2020 were used [21] and data used in this manuscript were assessed on 11/21/2021. All participants have written informed contents and no minor participants were enrolled. Participants enrolled in this trial had moderate depression and were randomized to receive either escitalopram or placebo (Please see more information such as inclusion and exclusion criteria, CONSORT diagram and treatment schema in [21]). Prior to treatment, all participants received structural magnetic resonance imaging (MRI). A total of 612 features, such as thickness or volume of 68 different brain regions, were extracted using a software program called Freesurfer [22]. Participants also filled out multiple questionnaires before treatment and provided other demographic and clinical information such as age, body mass index (BMI), comorbidities (other diseases) and age of MDD onset. Eight questionnaires were included in our study because they contained features that other research groups found to be useful in predicting antidepressant treatment responses based on our literature review. Hence we included all specific item scores, established domain/factor scores and total scores from these questionnaires as possible predictors in the later ML modeling step (Table 1). In addition, study participants were assessed by the Hamilton Depression Rating Scale (HDRS) longitudinally during 8 weeks of treatment on weeks 1, 2, 3, 4, 6 and 8.

**Table 1:** List of candidate features in APAT study for predicting remission.

MRI data obtained before treatment initiation (# of features = 657)	Cortical thickness, gray matter volume, folding index and other measures about the curvature and surface areas of 68 brain regions (34 left regions and 34 right regions), etc.
Questionnaire data administered at baseline containing possible predictors based on existing literature (# of features = 49, 36 HDRS and 13 QIDS)	1. HDRS: Hamilton Depression Rating Scale 2. QIDS: Quick Inventory of Depressive Symptoms Note: only listed the questionnaires that were used in both APAT study and EMBARC study.
Clinical and demographic data (# of features = 28)	age, education, sex, current medications, etc.

Remission was defined by a 17-item HDRS score of no more than seven after eight weeks of treatment [23]. Seven out of the 85 participants had missing week 8 HDRS Scores and hence their remission status could not be defined. The remission rate is 37.2% (29 out of 78) in the remaining 78 participants. There was no significant difference in the remission rate between participants in two treatment arms: 34.2% (13 out of 38) among participants treated with SSRI and 40% (16 out of 40) among participants treated with placebo (p-value = 0.6483). This is consistent with previous studies showing placebo response is similar to SSRI response neurobiologically [24] and clinically [25–28]. Relatedly, in the APAT cohort, neurobiological changes with treatment [29, 30] or prediction of treatment response [21, 31, 32] were not significantly affected by treatment type. Therefore, we pooled all participants from both treatment arms together to predict remission and included treatment assignment as a possible predictor. Table 2 described these 78 participants’ summary characteristics and questionnaire features by their known remission status (remission vs non-remission), where the same features that existed in both APAT study and EMBARC study (our external validation data)-were shown and a detailed questionnaire data dictionary is in Table A1 of S1 File.

**Table 2:** Descriptive statistics of study participants’ clinical variables, demographic variables and baseline questionnaire scores by remission status in APAT study.

Variable	N of participants with missing data	Level	Total	Non-Remitter (N = 49)	Remitter (N = 29)	P-value*
**Clinical and Demographic Features**						
age (years)	0	49 vs 29	23.66±13.12	23.35±10.09	22.57±13.78	0.5626
Total education years	8	49 vs 28	15.00±2.00	15.00±2.50	14.50±3.00	0.0664
sex	0	F	56 (65.88%)	36 (73.47%)	17 (58.62%)	0.2068
		M	29 (34.12%)	13 (26.53%)	12 (41.38%)	
Treatment	0	SSRI	42 (49.41%)	25 (51.02%)	13 (44.83%)	0.6483
		placebo	43 (50.59%)	24 (48.98%)	16 (55.17%)	
**QIDS**						
QIDS_01 (Falling Asleep)	4	49 vs 25	2.00±2.00	2.00±2.00	2.00±2.00	0.9715
QIDS_02 (Sleep During the Night)	4	49 vs 25	2.00±2.00	2.00±2.00	2.00±1.00	0.3711
QIDS_03 (Waking up Too Early)	4	49 vs 25	1.00±2.00	1.00±2.00	0.00±1.00	0.2559
QIDS_04 (Sleeping Too Much)	4	49 vs 25	0.00±1.00	0.00±1.00	0.00±1.00	0.4725
QIDS_05 (Feeling Sad)	4	49 vs 25	2.00±1.00	2.00±1.00	2.00±1.00	0.0209
QIDS_10 (Concentration/decision making)	4	49 vs 25	2.00±1.00	2.00±1.00	2.00±1.00	0.1869
QIDS_11 (View of Myself)	4	49 vs 25	2.00±2.00	3.00±2.00	1.00±2.00	0.0637
QIDS_12 (Thoughts of Death or Suicide)	4	49 vs 25	1.00±1.00	1.00±1.00	1.00±1.00	0.0998
QIDS_13 (General Interest)	4	49 vs 25	1.00±1.00	1.00±1.00	1.00±1.00	0.5220
QIDS_14 (Energy Level)	4	49 vs 25	2.00±1.00	2.00±1.00	2.00±1.00	0.1115
QIDS_15 (Feeling slowed down)	4	49 vs 25	1.00±1.00	1.00±1.00	1.00±1.00	0.0046
QIDS_16 (Feeling Restless)	4	49 vs 25	1.00±1.00	1.00±1.00	1.00±1.00	0.5209
QIDS total score	4	49 vs 25	15.00±5.00	15.00±5.00	13.00±5.00	0.0384
**HDRS**						
**Factor 1: Psychic depression**						
HDRS_01 (Depressed mood)	0	49 vs 29	2.00±2.00	2.00±2.00	2.00±1.00	0.6154
HDRS_02 (Feelings of guilt)	0	49 vs 29	2.00±1.00	2.00±1.00	2.00±1.00	0.0499
HDRS_03 (Suicide)	0	49 vs 29	1.00±2.00	1.00±2.00	0.00±1.00	0.0132
HDRS_08 (Retardation)	0	0	68 (80.00%)	41 (83.67%)	20 (68.97%)	0.1899
		1	14 (16.47%)	6 (12.24%)	8 (27.59%)	
		2	3 (3.53%)	2 (4.08%)	1 (3.45%)	
HDRS_22 (Helplessness)	0	49 vs 29	2.00±1.00	2.00±0.00	2.00±1.00	0.0039
HDRS_23 (Hopelessness)	0	49 vs 29	2.00±2.00	2.00±1.00	2.00±2.00	0.2418
HDRS_24 (Worthlessness)	0	49 vs 29	2.00±1.00	2.00±2.00	1.00±1.00	0.0275
HDRS_F1_AS	0	49 vs 29	11.00±5.00	11.00±5.00	9.00±3.00	0.0129
HDRS_F1_LWS	0	49 vs 29	6.30±2.97	6.58±3.53	5.29±2.35	0.0145
Note: a. HDRS_F1_AS was calculated by the arithmetic sum of all HDRS scores in Factor 1. b. HDRS_F1_LWS = 0.59* HDRS_01+0.46* HDRS_02+0.67* HDRS_03+0.44* HDRS_08+0.41* HDRS_22+0.65* HDRS_23+0.79* HDRS_24.						
**Factor 2: Loss of motivated behavior**						
HDRS_07 (Work and activities)	0	49 vs 29	2.00±1.00	2.00±1.00	2.00±1.00	0.2583
HDRS_12 (Somatic symptoms (appetite))	0	0	44 (51.76%)	26 (53.06%)	15 (51.72%)	0.9037
		1	23 (27.06%)	13 (26.53%)	9 (31.03%)	
		2	18 (21.18%)	10 (20.41%)	5 (17.24%)	
HDRS_14 (Genital symptoms (libido))	0	0	46 (54.12%)	31 (63.27%)	13 (44.83%)	0.2107
		1	17 (20.00%)	7 (14.29%)	8 (27.59%)	
		2	22 (25.88%)	11 (22.45%)	8 (27.59%)	
HDRS_16 (Weight loss)	0	0	76 (89.41%)	44 (89.80%)	25 (86.21%)	0.7343
		1	3 (3.53%)	1 (2.04%)	2 (6.90%)	
		2	6 (7.06%)	4 (8.16%)	2 (6.90%)	
HDRS_F2_AS	0	49 vs 29	4.00±3.00	4.00±3.00	4.00±3.00	0.8784
HDRS_F2_LWS	0	49 vs 29	2.10±1.34	2.10±1.26	2.18±1.68	0.8438
Note: a. HDRS_F2_AS was calculated by the arithmetic sum of all HDRS scores in Factor 2. b. HDRS_F2_LWS = 0.42* HDRS_07+0.84* HDRS_12+0.50* HDRS_14+0.74* HDRS_16.						
**Factor 3: Psychosis**						
HDRS_17 (Insight)	0	0	85 (100.00%)	49 (100.00%)	29 (100.00%)	.
HDRS_19 (Depersonalization and derealization)	0	0	75 (88.24%)	40 (81.63%)	28 (96.55%)	0.1500
		1	6 (7.06%)	5 (10.20%)	1 (3.45%)	
		2	4 (4.71%)	4 (8.16%)	0 (0.00%)	
HDRS_20 (Paranoid symptoms)	0	0	80 (94.12%)	44 (89.80%)	29 (100.00%)	0.2145
		1	4 (4.71%)	4 (8.16%)	0 (0.00%)	
		2	1 (1.18%)	1 (2.04%)	0 (0.00%)	
HDRS_21 (Obsessive and compulsive)	0	0	76 (89.41%)	45 (91.84%)	26 (89.66%)	0.1718
		1	6 (7.06%)	4 (8.16%)	1 (3.45%)	
		2	3 (3.53%)	0 (0.00%)	2 (6.90%)	
HDRS_F3_AS	0	49 vs 29	0.00±0.00	0.00±1.00	0.00±0.00	0.0561
HDRS_F3_LWS	0	49 vs 29	0.00±0.00	0.00±0.41	0.00±0.00	0.0633
Note: a. HDRS_F3_AS was calculated by the arithmetic sum of all HDRS scores in Factor 3. b. HDRS_F3_LWS = 0.74* HDRS_17+0.41* HDRS_19+0.68* HDRS_20+0.68* HDRS_21.						
**Factor 4: Anxiety**						
HDRS_09 (Agitation)	0	49 vs 29	1.00±1.00	1.00±1.00	0.00±1.00	0.1640
HDRS_10 (Anxiety—psychic)	0	49 vs 29	2.00±2.00	2.00±1.00	2.00±1.00	0.0016
HDRS_11 (Anxiety—somatic)	0	49 vs 29	1.00±1.00	2.00±1.00	1.00±2.00	0.0102
HDRS_15 (Hypochondrias)	0	49 vs 29	0.00±1.00	0.00±1.00	0.00±1.00	0.1912
HDRS_F4_AS	0	49 vs 29	5.00±2.00	5.00±2.00	4.00±2.00	0.0004
HDRS_F4_LWS	0	49 vs 29	2.90±1.58	3.08±1.58	2.28±1.42	0.0009
Note: a. HDRS_F4_AS was calculated by the arithmetic sum of all HDRS scores in Factor 4. b. HDRS_F4_LWS = 0.74* HDRS_19+0.62* HDRS_10+0.52* HDRS_11+0.68* HDRS_15.						
**Factor 5: Sleep disturbance**						
HDRS_04 (Insomnia—early)	0	0	32 (37.65%)	17 (34.69%)	11 (37.93%)	0.5061
		1	7 (8.24%)	3 (6.12%)	4 (13.79%)	
		2	46 (54.12%)	29 (59.18%)	14 (48.28%)	
HDRS_05 (Insomnia—middle)	0	0	31 (36.47%)	21 (42.86%)	9 (31.03%)	0.5626
		1	27 (31.76%)	14 (28.57%)	11 (37.93%)	
		2	27 (31.76%)	14 (28.57%)	9 (31.03%)	
HDRS_06 (Insomnia—late)	0	0	47 (55.29%)	23 (46.94%)	19 (65.52%)	0.2884
		1	16 (18.82%)	11 (22.45%)	4 (13.79%)	
		2	22 (25.88%)	15 (30.61%)	6 (20.69%)	
HDRS_F5_AS	0	49 vs 29	3.00±2.00	3.00±2.00	3.00±2.00	0.5036
HDRS_F5_LWS	0	49 vs 29	2.07±1.96	2.07±1.72	2.07±1.36	0.6934
Note: a. HDRS_F5_AS was calculated by the arithmetic sum of all HDRS scores in Factor 5. b. HDRS_F5_LWS = 0.74* HDRS_04+0.83* HDRS_05+0.59* HDRS_06.						
HDRS_13 (Somatic symptoms—General)	0	0	7 (8.24%)	1 (2.04%)	2 (6.90%)	0.6436
		1	29 (34.12%)	17 (34.69%)	11 (37.93%)	
		2	49 (57.65%)	31 (63.27%)	16 (55.17%)	
HDRS_18 (Diurnal variation)	0	49 vs 29	2.00±3.00	2.00±3.00	2.00±3.00	0.5327
HDRS 17 Baseline Total	0	49 vs 29	18.00±5.00	18.00±4.00	16.00±5.00	0.0049
*: For categorical variables, p-values were based on Chi-squared test with exact p-value from Monte Carlo simulation; for continuous variable, p-value was based on Wilcoxon rank sum test. Note: For continuous variable, median+/-IQR were reported. HDRS factor classifications and calculations of load-weighted sum from a 24-item HDRS were based on Milak *et al*., 2005.HDRS 17 Baseline Total was calculated by the sum of 17 HDRS scores from HDRS_01 to HDRS_17.						

### EMBARC study

The Establishing Moderators and Biosignatures of Antidepressant Response for Clinical Care for Depression (EMBARC) data were further included as an external validation dataset (ClinicalTrials.gov, NCT01407094; U01 MH092250, http://embarc.utsouthwestern.edu/). In this study, 296 participants with MDD were enrolled in a 2-stage Sequential Multiple Assignment Randomized Trial (SMART) design in 4 different centers including Massachusetts General Hospital Boston (MG), University of Michigan Ann Arbor (UM), Columbia University New York City (CU), and UT Southwestern Medical Center Dallas (TX). Study participants were assigned to two treatment arms: SSRI or placebo. Details on the EMBARC study design can be found in [33]. The deidentified EMBARC data are available through the NIH repository (https://nda.nih.gov/edit_collection.html?id=2199). We downloaded the data on 6/1/2018 and we did not have access to identifying information. Baseline features including neuroimaging, neurophysiological, and behavioral moderators from 197 participants were extracted for analysis in this study. However, 50 of them did not complete the 17-item HDRS at week 8. Table 3 described these 147 participants with observed HDRS week 8 score and their characteristics and questionnaire features by their known remission status (remitter vs non-remitter).

**Table 3:** Descriptive statistics of study participants’ clinical variables, demographic variables and baseline questionnaire scores by remission status in EMBARC study.

Variable	N missing	Level	Total	Non-Remitter (N = 92)	Remitter (N = 55)	P-value*
**Clinical and Demographic Features**						
Age (years)	0	92 vs 55	33.00±22.00	35.00±23.00	33.00±22.00	0.6223
total education years	3	91 vs 53	15.00±3.00	15.00±4.00	16.00±2.25	0.6565
sex	2	F	124 (63.59%)	53 (58.24%)	37 (68.52%)	0.2983
		M	71 (36.41%)	38 (41.76%)	17 (31.48%)	
treatment	0	SSRI	94 (47.72%)	43 (46.74%)	24 (43.64%)	0.7147
		Placebo	103 (52.28%)	49 (53.26%)	31 (56.36%)	
**QIDS**						
QIDS_01 (Falling Asleep)	0	92 vs 55	2.00±1.00	2.00±1.00	2.00±1.00	0.4469
QIDS_02 (Sleep During the Night)	0	92 vs 55	2.00±2.00	2.00±2.00	2.00±2.00	0.4470
QIDS_03 (Waking up Too Early)	0	92 vs 55	1.00±2.00	1.00±2.50	1.00±2.00	0.0610
QIDS_04 (Sleeping Too Much)	0	92 vs 55	1.00±2.00	1.00±2.00	1.00±2.00	0.9201
QIDS_05 (Feeling Sad)	0	1	10 (5.08%)	5 (5.43%)	2 (3.64%)	0.0426
		2	89 (45.18%)	34 (36.96%)	32 (58.18%)	
		3	98 (49.75%)	53 (57.61%)	21 (38.18%)	
QIDS_10 (Concentration/decision making)	0	92 vs 55	2.00±0.00	2.00±0.00	2.00±0.00	0.3135
QIDS_11 (View of Myself)	0	92 vs 55	3.00±1.00	3.00±1.00	3.00±1.00	0.8812
QIDS_12 (Thoughts of Death or Suicide)	1	91 vs 55	1.00±2.00	1.00±1.00	1.00±2.00	0.1639
QIDS_13 (General Interest)	1	91 vs 55	2.00±1.00	2.00±1.00	2.00±2.00	0.2943
QIDS_14 (Energy Level)	1	91 vs 55	2.00±0.00	2.00±0.00	2.00±0.00	0.1003
QIDS_15 (Feeling slowed down)	1	91 vs 55	1.00±1.00	1.00±1.00	1.00±1.00	0.8498
QIDS_16 (Feeling Restless)	1	91 vs 55	1.00±2.00	1.00±1.00	1.00±2.00	0.0498
QIDS_total	1	91 vs 55	18.00±4.00	18.00±4.00	18.00±6.00	0.7653
HDRS						
**Factor 1: Psychic depression**						
HDRS_01 (Depressed mood)HDRS	2	91 vs 54	3.00±1.00	3.00±1.00	2.50±1.00	0.5529
HDRS_02 (Feelings of guilt)	2	91 vs 54	2.00±1.00	2.00±1.00	2.00±1.00	0.8379
HDRS_03 (Suicide)	2	91 vs 54	3.00±1.00	3.00±1.00	2.00±1.00	0.1121
HDRS_08 (Retardation)	2	91 vs 54	1.00±2.00	1.00±2.00	0.00±2.00	0.4721
HDRS_22 (Helplessness)	2	91 vs 54	2.00±1.00	2.00±0.00	2.00±1.00	0.2130
HDRS_23 (Hopelessness)HDRS	2	91 vs 54	1.00±2.00	1.00±2.00	1.00±2.00	0.2168
HDRS_24 (Worthlessness)	2	91 vs 54	2.00±1.00	2.00±1.00	2.00±1.00	0.6532
HDRS_F1_AS	2	91 vs 54	13.00±4.00	13.00±3.00	13.00±4.00	0.3739
HDRS_F1_LWS	2	91 vs 54	7.29±2.40	7.25±2.17	7.56±2.08	0.4076
Note: a. HDRS_F1_AS was calculated by the arithmetic sum of all HDRS scores in Factor 1. b. HDRS_F1_LWS = 0.59* HDRS_01+0.46* HDRS_02+0.67* HDRS_03+0.44* HDRS_08+0.41* HDRS_22+0.65* HDRS_23+0.79* HDRS_24.						
**Factor 2: Loss of motivated behavior**						
HDRS_07 (Work and activities)	2	91 vs 54	1.00±2.00	1.00±2.00	1.00±1.00	0.0462
HDRS_12 (Somatic symptoms (appetite))	2	91 vs 54	0.00±1.00	0.00±1.00	0.00±1.00	0.2176
HDRS_14 (Genital symptoms (libido))	2	91 vs 54	0.00±1.00	0.00±0.00	0.00±1.00	0.0081
HDRS_16 (Weight loss)	2	91 vs 54	0.00±1.00	0.00±1.00	0.00±0.00	0.0721
HDRS_F2_AS	2	91 vs 54	2.00±2.00	2.00±2.00	2.00±2.00	0.1594
HDRS_F2_LWS	2	91 vs 54	1.16±1.42	1.26±1.16	0.92±1.00	0.1497
Note: a. HDRS_F2_AS was calculated by the arithmetic sum of all HDRS scores in Factor 2. b. HDRS_F2_LWS = 0.42* HDRS_07+0.84* HDRS_12+0.50* HDRS_14+0.74* HDRS_16.						
**Factor 3: Psychosis**						
HDRS_17 (Insight)	57	71 vs 32	0.00±0.00	0.00±0.00	0.00±0.00	0.8969
HDRS_19 (Depersonalization and derealization)	2	91 vs 54	3.00±2.00	3.00±2.00	3.00±1.00	0.2487
HDRS_20 (Paranoid symptoms)	2	91 vs 54	1.00±2.00	1.00±2.00	1.00±2.00	0.2337
HDRS_21 (Obsessive and compulsive)	2	91 vs 54	2.00±1.00	2.00±1.00	2.00±1.00	0.6382
HDRS_F3_AS	57	71 vs 32	6.00±2.00	6.00±3.00	6.00±2.50	0.9914
HDRS_F3_LWS	57	71 vs 32	3.27±1.69	3.27±1.77	3.14±1.72	0.8499
Note: a. HDRS_F3_AS was calculated by the arithmetic sum of all HDRS scores in Factor 3. b. HDRS_F3_LWS = 0.74* HDRS_17+0.41* HDRS_19+0.68* HDRS_20+0.68* HDRS_21.						
**Factor 4: Anxiety**						
HDRS_09 (Agitation)	2	91 vs 54	0.00±0.00	0.00±0.00	0.00±1.00	0.5704
HDRS_10 (Anxiety—psychic)	2	91 vs 54	1.00±1.00	0.00±1.00	1.00±1.00	0.0982
HDRS_11 (Anxiety—somatic)	2	91 vs 54	0.00±1.00	0.00±1.00	0.00±0.00	0.0212
HDRS_15 (Hypochondrias)	53	72 vs 33	0.00±0.00	0.00±0.00	0.00±0.00	0.6377
HDRS_F4_AS	53	72 vs 33	1.00±2.00	1.00±1.00	1.00±1.00	0.8578
HDRS_F4_LWS	53	72 vs 33	0.74±1.46	0.62±0.90	0.62±0.84	0.6904
Note: a. HDRS_F4_AS was calculated by the arithmetic sum of all HDRS scores in Factor 4. b. HDRS_F4_LWS = 0.74* HDRS_19+0.62* HDRS_10+0.52* HDRS_11+0.68* HDRS_15.						
**Factor 5: Sleep disturbance**						
HDRS_04 (Insomnia—early)	2	91 vs 54	2.00±1.00	2.00±1.00	2.00±1.00	0.7927
HDRS_05 (Insomnia—middle)	2	91 vs 54	2.00±1.00	3.00±1.00	2.00±2.00	0.0242
HDRS_06 (Insomnia—late)	2	91 vs 54	1.00±2.00	2.00±2.00	1.00±2.00	0.3630
HDRS_F5_AS	2	91 vs 54	5.00±2.00	5.00±3.00	5.00±3.00	0.1139
HDRS_F5_LWS	2	91 vs 54	3.97±1.66	3.97±1.92	3.66±2.07	0.1006
Note: a. HDRS_F5_AS was calculated by the arithmetic sum of all HDRS scores in Factor 5. b. HDRS_F5_LWS = 0.74* HDRS_04+0.83* HDRS_05+0.59* HDRS_06.						
HDRS_13 (Somatic symptoms—General)	2	91 vs 54	1.00±2.00	1.00±2.00	0.00±1.00	0.7050
HDRS_18 (Diurnal variation)	2	91 vs 54	2.00±1.00	2.00±1.00	2.00±1.00	0.4203
HDRS 17 Baseline Total	60	69 vs 31	19.00±6.00	18.00±6.00	18.00±6.00	0.4197
*: For categorical variables, p-values were based on Chi-squared test with exact p-value from Monte Carlo simulation; for continuous variable, p-value was based on Wilcoxon rank sum test. Note: For continuous variable, median+/-IQR were reported. HDRS factor classifications and calculations of load-weighted sum were from 24-item HDRS based on Milak *et al*., 2005. HDRS 17 Baseline Total was calculated by the sum of 17 HDRS scores from HDRS_01 to HDRS_17.						

### Data pre-processing and feature selection

**Fig 1:**
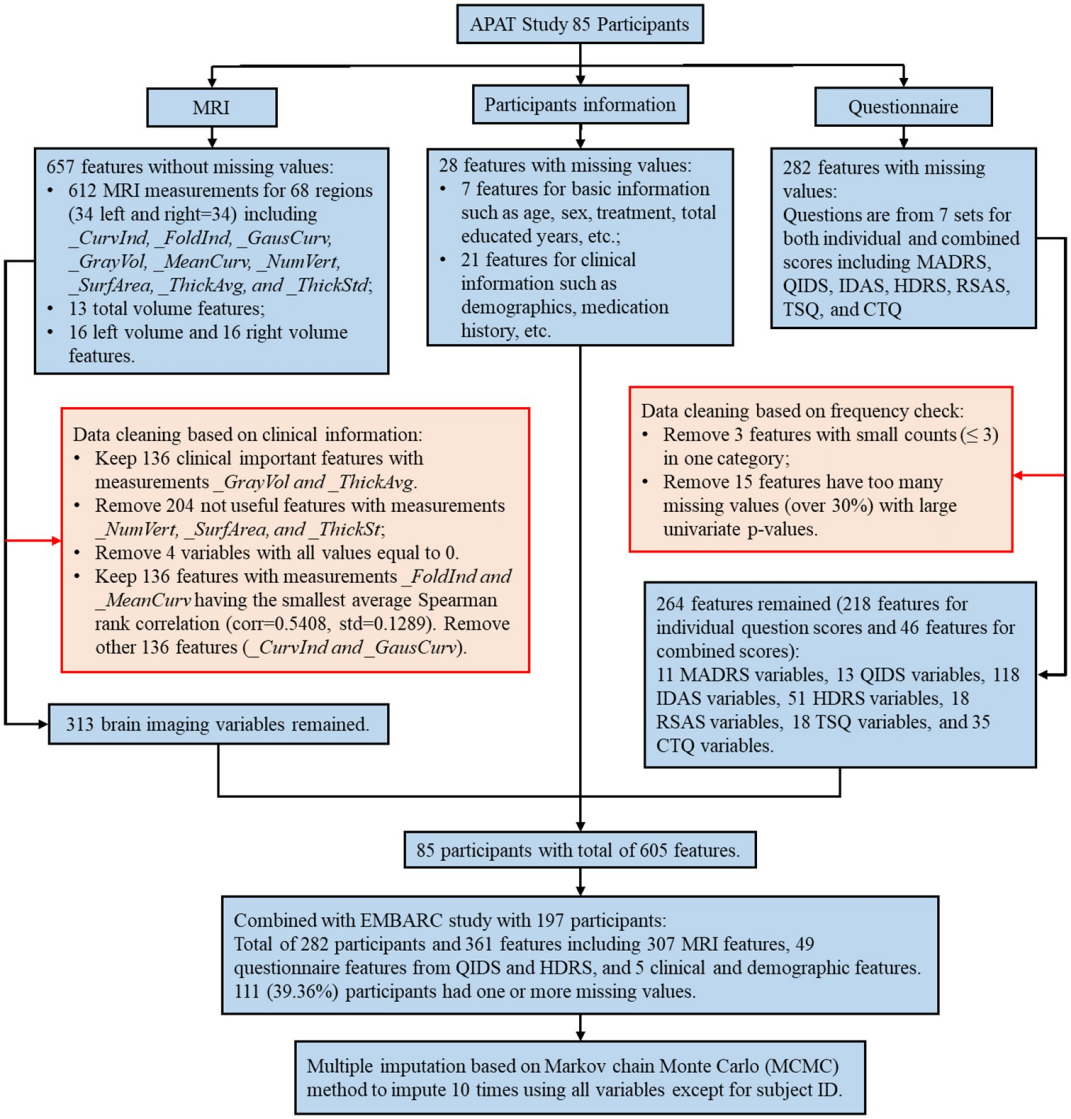
Flowchart of data pre-processing.

Since we planned to use EMBARC data as an external validation set to examine our proposed ML pipeline for APAT data, we first cleaned the APAT data based on clinical and statistical information and then kept the same features that existed in both APAT and EMBARC datasets. Fig 1 provides an overview of this step.

In the APAT study, the structural MRI data contained different measures from all 68 brain regions (34 left and 34 right brain regions). Besides regions’ gray volume and cortical thickness, other measures reported by Freesurfer included folding index, curvature index etc. To remove the redundancy and multicollinearity in these measures, we checked the pair-wise correlations using the average Spearman’s rank correlation coefficient from all 68 brain regions and only kept measures that are not highly correlated with each other (with an average correlation coefficient <0.7). Consequently, only 4 different measures (cortical thickness, gray matter volume, folding index and mean curvature) from each region were used in the following analyses (total number of features from MRI = 272). We further eliminated some more features whose information was included in multiple measures. For example, we included BMI, which is a combined measure of height and weight, but excluded individual height and weight. In addition, features with only one category or small counts (≤ 3) in one category or too many missing values were excluded. In the end, we have 605 features combining MRI, questionnaire, clinical and demographic data after these initial feature selection steps.

After keeping the same features of APAT data and EMBARC data, there were total of 282 participants with 361 features, where 307 brain imaging features, 49 questionnaire features from QIDS and HDRS (including baseline and week 8 HDRS scores), 4 clinical and demographic features (age, total years of education, sex, and medication use).

Among all 282 participants enrolled in both clinical trials, 111 (39.36%) had one or more missing values among the 361 features, 12 participants from APAT study and 99 participants from EMBARC study. 57 participants had missing week 8 HDRS scores (7 from APAT study and 50 from EMABRC study). To use all available data from these 282 participants and optimize the sample size for small dataset, multiple imputation based on Markov chain Monte Carlo (MCMC) method was used to create 10 new imputed datasets to eliminate missing data [34, 35]. The missing data were imputed through a random sampling from a plausible statistical model using other existing data in the dataset. Therefore, several different plausible datasets were generated in order to estimate the uncertainty about the missing data. We chose to generate 10 such imputed datasets as most papers used < = 10 imputations and actually 3–5 imputations could lead to excellent results in theory [36]. We used PROC MI in SAS 9.4 (SAS Institute Inc., Cary, NC) to impute all missing data. Imputed values for features that only had integer values were rounded and constrained within their existing lower and upper boundaries in the dataset. For example, if an imputed value was less than 0 but its lower bound was, it would be assigned a value of 0. Week-8 HDRS scores were imputed for the 57 participants who were missing them, and as such their missing remission status could then be determined. Table 4 showed the frequency of remission in all 10 imputed datasets ranging from 34.12% to 40% for APAT data and 30.46% to 39.09% for EMBARC data.

**Table 4:** Frequency table of remission vs non-remission in all 10 imputed datasets within each of two studies.

Imputation	EMBARC		APAT	
	Non-Remission N (%)	Remission N (%)	Non-Remission N (%)	Remission N (%)
1	120 (60.91%)	77 (39.09%)	54 (63.53%)	31 (36.47%)
2	134 (68.02%)	63 (31.98%)	56 (65.88%)	29 (34.12%)
3	137 (69.54%)	60 (30.46%)	54 (63.53%)	31 (36.47%)
4	127 (64.47%)	70 (35.53%)	55 (64.71%)	30 (35.29%)
5	122 (61.93%)	75 (38.07%)	54 (63.53%)	31 (36.47%)
6	124 (62.94%)	73 (37.06%)	54 (63.53%)	31 (36.47%)
7	129 (65.48%)	68 (34.52%)	56 (65.88%)	29 (34.12%)
8	121 (61.42%)	76 (38.58%)	51 (60%)	34 (40%)
9	124 (62.94%)	73 (37.06%)	54 (63.53%)	31 (36.47%)
10	120 (60.91%)	77 (39.09%)	56 (65.88%)	29 (34.12%)

For each imputed dataset, we first used APAT data as the training set and EMBARC data as the testing set. But to evaluate the effect on prediction performance due to possible study site heterogeneity, we also divided the 282 participants into 2/3 training set and 1/3 testing set where no systematic study site difference exists in addition to using EMBARC data as the training set and APAT data as the testing set.

Since the sample size was much smaller than the number of features, an appropriate feature selection method was essential instead of putting all the features into machine learning models. We considered various of feature selection methods including pairwise correlation (randomly kept one feature for the highly correlated pairs with correlation>0.7), univariate analysis (features were selected with a univariate p-value<0.1 based on Wilcoxon rank sum test for continuous features and Chi-squared test for categorical features by remission status), principal component analysis (PCA), and AutoEncoder [37]. However, none of the feature selection methods could obtain a relatively good performance (i.e., low accuracy or AUC). Therefore, according to the approaches that selected top 50 ranked features based on the importance ranking values [15, 38], we implemented a new method to select the top 50 most frequent top-ranked features based on our small dataset. 6 models including Random Forest, Lasso, Ridge, XGBoost, SVM, and Neural Network with 5 repeated 5-fold cross validation were iterated over the 10 imputed datasets. Top 20 ranked features were output each time so that we had a total of 30,000 (top 20 ranked features * repeated 5 times * 5-fold CV * 6 models * 10 datasets) features. Then we calculated the frequency for the above 300,000 features and kept the top 50 most frequent variables (Table A2 in S1 File has a list of these 50 features based on different training sets).

The importance ranking values describe how much each feature contributes to the prediction. The default importance ranking results from the Python ***sklearn*** module were used. For example, the importance ranking from the penalized logistic regression model is based on the fitted coefficients. For SVM and neural network approaches, which lack package-built-in feature importance measurements, permutation feature importance was used. Permutation feature importance is defined as the decrease of a model performance score when single feature values are randomly shuffled among participants, which can be implemented to any fitted predictors [39]. In Python, *permutation_importance* function from ***sklearn*.*inspection*** module was used to perform permutation feature importance ranking.

The last step before we constructed ML models was to standardize all continuous features by centering and scaling for each training and testing pairs from the cross-validation method using this formula: $\frac{x_{p}-\mu }{\sigma }$, where *x*_*p*_ denoted values of a feature/predictor, μ was the mean and σ was standard deviation of *x*_*p*_. This was to ensure that there was no bias within the data due to varying ranges of each feature and different unit. Standardization utilized the *StandardScaler*.*fit_transform* function in the Python module ***sklearn*.*preprocessing***.

### Predictive modeling

In APAT study, due to the relatively small number of participants in our dataset, we used five repeated four-fold CV to robustly evaluate the performance of our predictive modeling pipeline [40]. Basically, we partitioned the training data from an imputed dataset into four groups (folds) with relatively equal size of participants. Each fold was considered as the testing set and the remaining three folds were the training set used to train the ML models for predicting remission on the testing set. We repeated this process 5 times with different splits each time to minimize the variation across different split and increase the stability of the final results [41]. This modeling process used the function *RepeatedKFold* under Python module ***sklearn*.*model_selection***. To select the best set of hyperparameters for each of the four training sets, we further implemented a 3-fold nested cross-validation using function *GridSearchCV* under Python module ***sklearn*.*model_selection*** [40, 42, 43]. Furthermore, we applied our predictive modeling pipeline to an external dataset, the final predictor used the trained model based on 4-fold cross-validation but with all samples from the training data set.

For each training and testing split, following the feature selection (top 50 most frequent features) and standardization, 6 popularly usedML models based on Random Forest, Penalized Logistic Regression, XGBoost, Support Vector Machine (SVM), Gradient Boosting Decision Tree, and Neural Network were implemented. After ensembling the predicted results from the 6 models together, we further built a meta model named Stacking using logistic regression [42, 43].

1. Penalized logistic regression including *l1-regularization* Lasso regression [44], *l2-regularization* Ridge regression [45], and elastic-net regression [46] using *LogisticRegression* function under Python module ***sklearn*.*linear_model*** with parameter *penalty = "l1"/"l2"/"elasticnet"*.
2. Random forest [47] using *RandomForestClassifier* function from Python module ***sklearn*.*ensemble***.
3. Gradient boosted decision trees [48] using *GradientBoostingClassifier* function from Python module ***sklearn*.*ensemble***.
4. XGBoost [49] using *XGBClassifier* function in Python Python module ***xgboost***.
5. Support vector machine [50] using *SVC* function in the Python module ***sklearn*.*svm***.
6. Neural network [51] using *MLPClassifier* function from Python module ***sklearn*.*neural_network***.

Stacking method was an ensemble algorithm that combines results from base models (level 0 models) and then made prediction using the meta model (level 1 model). Based on the predicted probabilities from the above 6 models as base models each time, 6 features at level 1 modelling step were constructed for both training and testing sets, respectively. Logistic regression was further implemented as the meta model fitting with the 6 predictors to obtain the final predictive results. Logistic regression used *LogisticRegression* function under Python module ***sklearn*.*linear_model*** with parameter penalty = "none". Besides a stacking method using all 6 ML models, another stacking model was constructed using penalized logistic regression and neural network because of the better performance from these two base ML models on average in our study.

### Predictive performance evaluation

Two sets of model performance results including results from training set and results from testing set were reported. Model performances were assessed using measures such as accuracy defined as the % of all participants correctly classified, balanced accuracy (average of accuracy within remitters and non-remitters), area under the curve (AUC) which is a measure of discrimination, sensitivity (% of true positive), specificity (% of true negative), positive predictive value (% of true positive among positive predicted), and negative predictive value (% of true negative among positive predicted, [40]). The average and standard error were calculated for each performance measure. For the training set, the corresponding standard errors (se) were the combined results from 5 repeated 4-fold CV iterating over 10 imputed datasets, which were summarized using Rubin’s rule [34] and were used to calculate Wald-type 95% confidence intervals (CI: point estimate±1.96*se, see A0 in S1 File for detailed formula). For the testing set, the standard errors for testing set performances were calculated based on the formula $\sigma /\sqrt{n}$ (n = 10) for the 10 imputed datasets. The AUC values were calculated used function *roc_auc_score* under Python module ***sklearn*.*metrics*** and other performances were calculated from the confusion matrix using function *confusion_matrix* under Python module ***sklearn*.*metrics***.

ROC curves (sensitivity vs 1-specificity) for multiple sub-groups were generated based on the average results of testing sets from 10 imputed datasets using the ML model with the best predictive performance using internal cross-validation on training set. The steps are described below:

- Step 1: Divide the data first by subgroup then by imputation, and set a sequence of thresholds (e.g. seq(0, 1, -0.0001) or a vector of predicted probabilities).
- Step 2: Iterate over the thresholds and calculate the corresponding sensitivity and specificity for each single dataset.
- Step 3: Calculate the AUC for each single dataset.
- Step 4: Iterate over 10 imputed datasets to repeat Step 2 to Step 3, calculate the average sensitivity and average specificity by the sequence of thresholds, and calculate the average AUC over 10 imputed datasets.
- Step 5: Iterate over groups (sex, baseline_HDRS17, baseline_HDRS_F4_AS, baseline_HDRS_F4_LWS, placebo, site) and repeat Step 2 to Step 4.
- Step 6: Draw ROC curves by subgroup.

R function *compareROCindep()* from **nsROC** package was used to compare k (k> = 2) independent ROC curves for each imputed dataset, where the default setting of statistics based on nonparametric statistics named “L1 statistic proposed in Martinez-Camblor” was used [52]. After iterating over 10 imputed datasets, a list of 10 p-values were output. Harmonic mean p-value (HMP) method [53] was further implemented to combine the p-values assuming the hypothesis tests among 10 imputed datasets were dependent with unknown dependency structure. The null hypothesis for HMP is none of the p-values are significant. The formula of HMP to get final p-value, $\tilde{p}$, is

$$\tilde{p}=\frac{\sum_{i=1}^{L}\omega_{i}}{\sum_{i=1}^{L}\omega_{i}/p_{i}},$$

where *ω*_1_, *ω*_2_, …, *ω*_*L*_ are weights satisfying $\sum_{i=1}^{L}\omega_{i}=1$, where *p*_*i*_’*s* are the p-values for the imputed datasets, *L* is the total number of p-values, and *ω*_*i*_ = 1/*L* were used for our results. Example R codes can be found in section A4 of S1 File.

**Fig 2:**
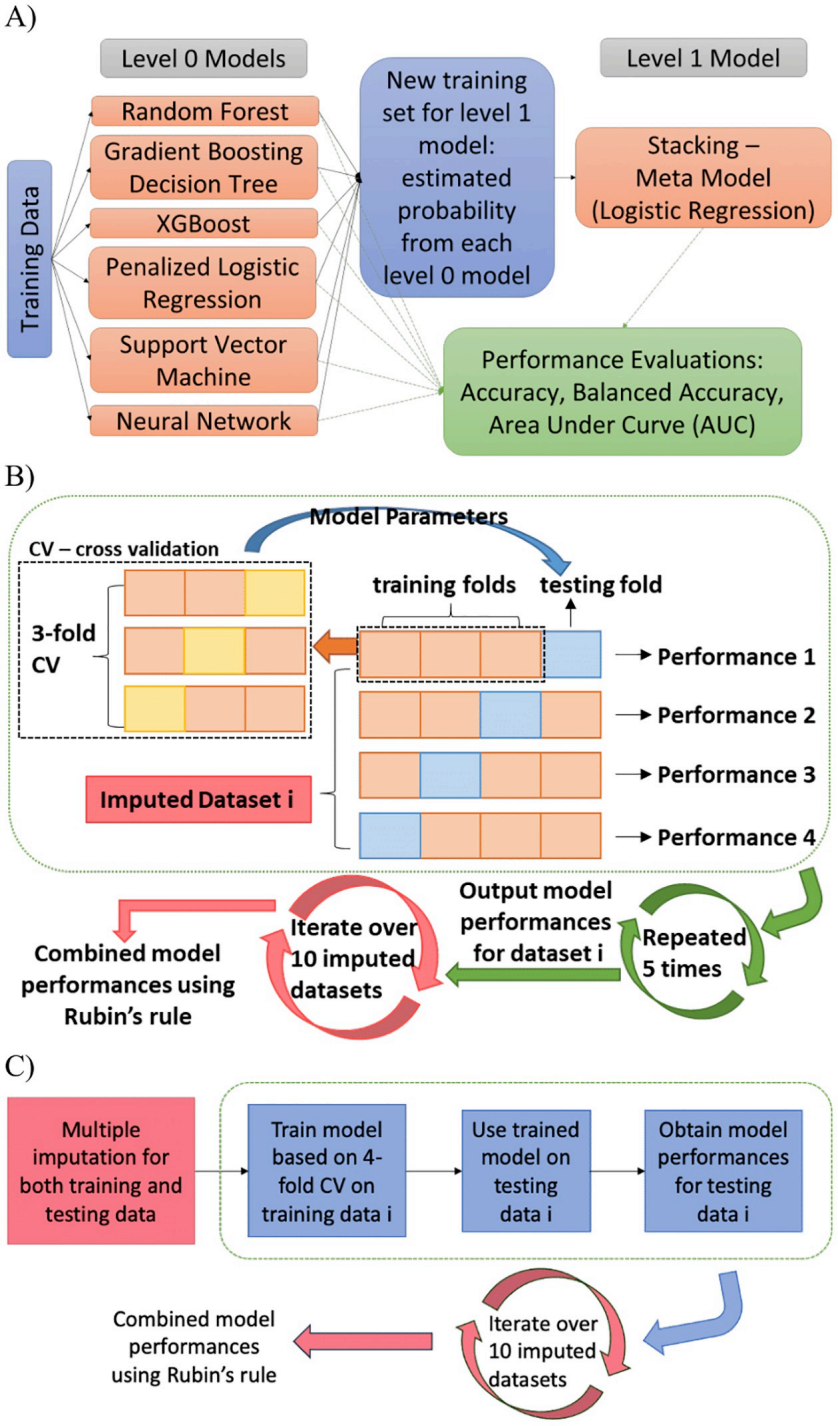
Overall predictive modeling pipeline. A) Stacking method: Predictions from six individual ML models were the inputs to construct the final predictive model; B) Predictive modeling pipeline from data preprocessing to final performance evaluation for training data only; C) Predictive modeling pipeline with external validation using testing data.

Fig 2 below illustrates our overall predictive modeling pipeline. Example python codes can be found in section A5 of S1 File.

## Results

### Using APAT data for training and EMBARC data for external validation

We considered the APAT data as training dataset and obtained the model performance evaluations in Table 5 including Sensitivity, Specificity, Positive predicted value, Negative predicted value, Accuracy, Balanced Accuracy, and AUC for binary outcome Remission. EMBARC data were used as the external validation set to examine the performance of our ML pipeline. According to the results, the penalized logistic regression model had the highest accuracy (0.8341 ± 0.0202) and AUC (0.8257 ± 0.0257) values among all 6 models plus 2 stacking models using different base ML models.

**Table 5:** Model performance results based on random forest, gradient boosting, penalized logistic regression, XGBoost, SVM, neural network, and stacking for APAT data as training set and EMBARC data as testing set after multiple imputation for 10 times.

Model	Name	sensitivity (se)	specificity (se)	PPV (se)	NPV (se)	ACC (se)	BA (se)	AUC (se)
Random Forest	Training set APAT	0.4305 (0.0577)	0.9582 (0.021)	0.8606 (0.0517)	0.7503 (0.009)	0.7692 (0.0145)	0.6944 (0.0259)	0.7054 (0.0298)
	Testing set EMBARC	0.4065 (0.1511)	0.5818 (0.1548)	0.3589 (0.072)	0.6304 (0.0538)	0.5188 (0.0614)	0.4941 (0.039)	0.4941 (0.0404)
Gradient Boosting	Training set APAT	0.2423 (0.0602)	0.9837 (0.0154)	0.9111 (0.0854)	0.6986 (0.0161)	0.7174 (0.0203)	0.613 (0.0299)	0.615 (0.0309)
	Testing set EMBARC	0.2945 (0.2883)	0.6859 (0.2928)	0.3229 (0.104)	0.6293 (0.0525)	0.5391 (0.1079)	0.4902 (0.0399)	0.4902 (0.041)
SVM	Training set APAT	0.6167 (0.052)	0.9363 (0.0158)	0.8477 (0.03)	0.814 (0.0136)	0.8219 (0.0141)	0.7765 (0.0238)	0.7824 (0.0325)
	Testing set EMBARC	0.6423 (0.0848)	0.3344 (0.0773)	0.3535 (0.0488)	0.6208 (0.079)	0.4467 (0.0395)	0.4884 (0.0379)	0.4884 (0.039)
XGBoost	Training set APAT	0.5688 (0.0529)	0.8745 (0.0245)	0.7205 (0.0487)	0.7838 (0.0139)	0.7652 (0.0208)	0.7216 (0.0276)	0.7256 (0.0302)
	Testing set EMBARC	0.4568 (0.1446)	0.5292 (0.1211)	0.3508 (0.0686)	0.6319 (0.0656)	0.5035 (0.0505)	0.493 (0.0462)	0.493 (0.047)
Penalized logistic regression	Training set APAT	0.7893 (0.0289)	0.8592 (0.0211)	0.7612 (0.023)	0.8786 (0.0213)	0.8341 (0.0202)	0.8243 (0.0202)	0.8257 (0.0257)
	Testing set EMBARC	0.7516 (0.0574)	0.2287 (0.0653)	0.3556 (0.0509)	0.6182 (0.0905)	0.4178 (0.0476)	0.4902 (0.0433)	0.4902 (0.044)
Neural Network	Training set APAT	0.7535 (0.0207)	0.8524 (0.0282)	0.7442 (0.0257)	0.8602 (0.0155)	0.8172 (0.0192)	0.8029 (0.0154)	0.8041 (0.0217)
	Testing set EMBARC	0.7744 (0.0665)	0.2021 (0.0708)	0.3548 (0.05)	0.6091 (0.0835)	0.4086 (0.0457)	0.4883 (0.0379)	0.4882 (0.039)
Stacking (Using above 6 models)	Training set APAT	0.5597 (0.0408)	0.9502 (0.0168)	0.8684 (0.0368)	0.7938 (0.013)	0.8101 (0.0154)	0.7549 (0.0201)	0.7554 (0.0279)
	Testing set EMBARC	0.5808 (0.149)	0.4043 (0.1531)	0.3547 (0.0584)	0.6253 (0.0667)	0.466 (0.0592)	0.4926 (0.0411)	0.4925 (0.0423)
Stacking (Using Penalized logistic regression and Neural Network)	Training set APAT	0.7515 (0.0198)	0.8546 (0.0296)	0.747 (0.0278)	0.8595 (0.0156)	0.8179 (0.02)	0.8031 (0.0156)	0.8045 (0.021)
	Testing set EMBARC	0.777 (0.0664)	0.2013 (0.0683)	0.3554 (0.0495)	0.611 (0.0838)	0.4091 (0.0446)	0.4892 (0.0376)	0.4892 (0.0389)

Note:

1. Average (se) were calculated for the following model performance: Sensitivity = sensitivity (True Positive Rate, Recall) = TP/(TP+FN); Specificity = specificity (True Negative Rate) = TN/(TN+FP); PPV (Positive Predicted Value, Precision) = TP/(TP+FP); NPV (Negative Predicted Value) = TN/(TN+FN); Accuracy (ACC) = (TP+TN)/(TP+TN+FP+FN); Balanced Accuracy (BA) = (sensitivity+specificity)/2

2. Standard error (se) was calculated based on Rubin’s rule for imputed data. For the se of AUC in testing data, bootstrapping method repeated 1000 times was used to estimate the within-subject variance.

**Fig 3:**
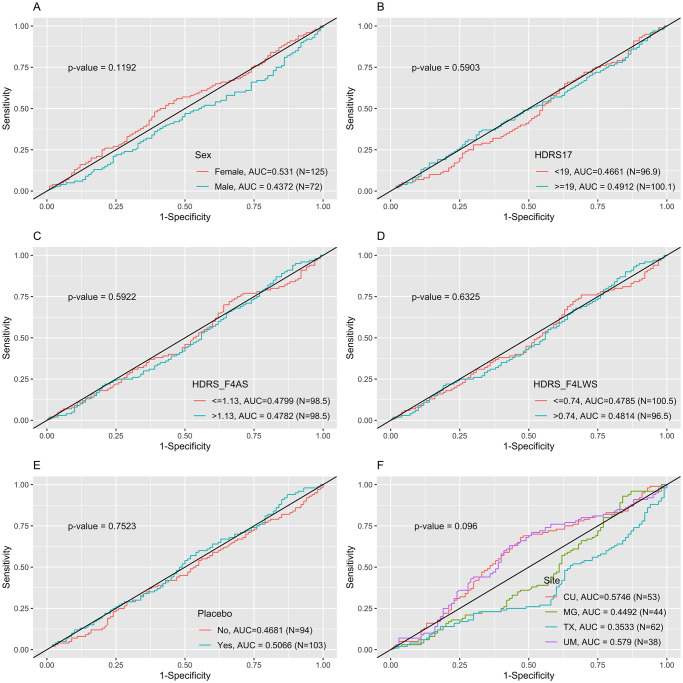
ROC curves comparison across groups based on penalized logistic regression which had the best predictive performance from internal cross-validation using training set.

However, the testing results via EMBARC data had much worse model performances, where all the AUC values were smaller than 0.5 indicating the models were meaningless. We note that the EMBARC data were from 4 different sites, while the APAT data were from the single site. In addition, the APAT study and EMBARC study provided different treatments. The APAT participants were significantly younger than EMBARC participants (median 23.66 vs. 33.00, p-value < 0.0001). Therefore, the ROC curves from predicting results of testing set (EMBARC data) using penalized logistic regression which had the best predictive performance from internal cross-validation using training set as suggested by Table 5 by different groups such as sex, HDRS 17, HDRS factor 4, medication or placebo, and site were drawn in Fig 3 to explore possible reasons for the unacceptable prediction performance while externally validating the ML model from APAT data. Even though all the p-values testing the differences between/among ROC curves were not significant for neither penalized logistic regression or stacking, we could still notice a difference of ROC curves by sites, where participants from CU and UM sites had higher AUC values than participants from TX and MG. To verify it is small sample size of training set or the site difference that caused the issue, we further performed another two sets of results using (1) EMBARC data as training set with sample sized doubled and APAT data as testing set, and (2) the 2/3 combined APAT and EMBARC data as training set and 1/3 combined data as testing set.

### Using EMBARC data for training data and APAT data as external validation data

**Fig 4:**
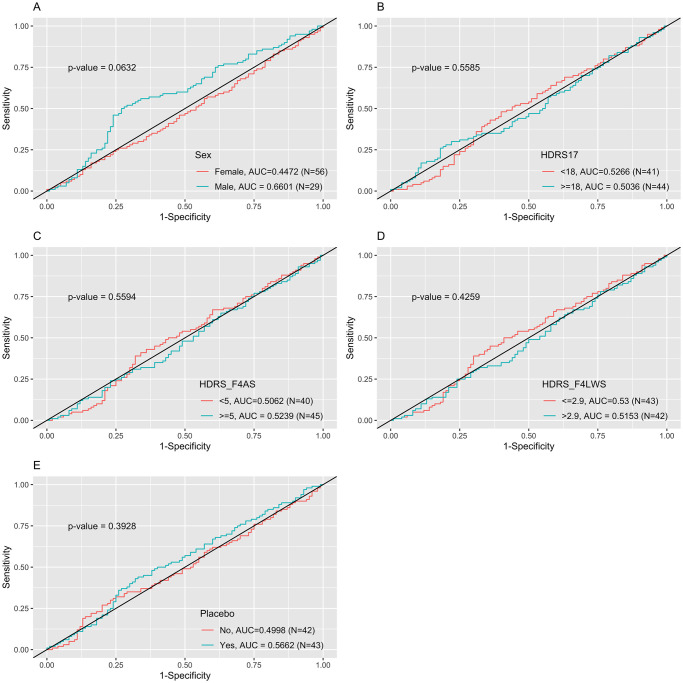
ROC curves comparison across groups based on the stacking method using penalized logistic regression and neural network which had the best predictive performance from internal cross-validation using training set.

Since EMBARC data had larger sample size than APAT data, we used EMBARC data as the training set and APAT data as the external validation set. Similar model performance results were shown in Table 6 and Fig 4. In Table 6, the stacking method with results from penalized logistic regression and neural network had the highest accuracy (0.7026 ± 0.0254) and AUC (0.6778 ± 0.0211) values among all 6 models plus 2 stacking models. The model performances using EMBARC data as training set had a significant drop comparing to the results using APAT as training set. Besides, the accuracies and AUCs using APAT data as testing set had a slightly increase than the above EMARC data as testing set (AUCs around 0.5).

**Table 6:** Model performance results based on random forest, gradient boosting, penalized logistic regression, XGBoost, SVM, neural network, and stacking for EMBARC data as training set and APAT data as testing set after multiple imputation for 10 times.

Model	Name	sensitivity (se)	specificity (se)	PPV (se)	NPV (se)	ACC (se)	BA (se)	AUC (se)
Random Forest	Training set EMBARC	0.1096 (0.0714)	0.9565 (0.0315)	0.6221 (0)	0.6557 (0.0263)	0.6524 (0.029)	0.5331 (0.0227)	0.5384 (0.0242)
	Testing set APAT	0.0126 (0.0324)	0.9759 (0.0576)	0.2583 (0.2154)	0.6371 (0.0566)	0.6294 (0.0619)	0.4942 (0.0571)	0.4942 (0.0491)
Gradient Boosting	Training set EMBARC	0.2463 (0.066)	0.8858 (0.032)	0.5516 (0.0698)	0.6759 (0.0314)	0.6557 (0.0313)	0.566 (0.0278)	0.5697 (0.0263)
	Testing set APAT	0.0871 (0.091)	0.8877 (0.0974)	0.3884 (0.3461)	0.6327 (0.0585)	0.6 (0.0605)	0.4874 (0.0595)	0.4874 (0.0516)
SVM	Training set EMBARC	0.3618 (0.0697)	0.8595 (0.0494)	0.6024 (0.0485)	0.7049 (0.0231)	0.6823 (0.0305)	0.6107 (0.0238)	0.6171 (0.0245)
	Testing set APAT	0.1335 (0.0804)	0.8875 (0.0577)	0.3914 (0.1855)	0.6455 (0.0601)	0.6165 (0.0599)	0.5105 (0.059)	0.5105 (0.0516)
XGBoost	Training set EMBARC	0.3939 (0.0522)	0.7678 (0.042)	0.4921 (0.043)	0.6915 (0.0287)	0.6345 (0.0303)	0.5808 (0.0227)	0.5875 (0.0225)
	Testing set APAT	0.1941 (0.1431)	0.7679 (0.1672)	0.3535 (0.1962)	0.627 (0.067)	0.56 (0.0857)	0.481 (0.0695)	0.481 (0.0634)
Penalized logistic regression	Training set EMBARC	0.56 (0.0493)	0.7744 (0.038)	0.5854 (0.0501)	0.7571 (0.0264)	0.6983 (0.0295)	0.6672 (0.0295)	0.6717 (0.0302)
	Testing set APAT	0.1753 (0.0946)	0.8286 (0.0604)	0.3592 (0.1515)	0.6414 (0.0617)	0.5941 (0.0598)	0.502 (0.062)	0.502 (0.0546)
Neural Network	Training set EMBARC	0.5663 (0.0369)	0.7778 (0.0359)	0.5933 (0.0289)	0.7598 (0.0281)	0.7026 (0.0268)	0.672 (0.0208)	0.6775 (0.022)
	Testing set APAT	0.23 (0.1112)	0.7926 (0.0783)	0.3764 (0.1301)	0.6474 (0.064)	0.5894 (0.0602)	0.5113 (0.0605)	0.5113 (0.0528)
Stacking (Using above 6 models)	Training set EMBARC	0.3892 (0.0576)	0.8457 (0.0331)	0.5915 (0.0378)	0.7105 (0.0283)	0.6822 (0.0279)	0.6174 (0.0231)	0.6242 (0.0229)
	Testing set APAT	0.1307 (0.0968)	0.8699 (0.0988)	0.4396 (0.2847)	0.6394 (0.0584)	0.6035 (0.0651)	0.5003 (0.0617)	0.5003 (0.0549)
Stacking (Using Penalized logistic regression and Neural Network)	Training set EMBARC	0.5676 (0.0359)	0.7771 (0.0344)	0.5929 (0.0285)	0.7603 (0.0273)	0.7026 (0.0254)	0.6724 (0.0196)	0.6778 (0.0211)
	Testing set APAT	0.23 (0.1112)	0.7944 (0.0768)	0.3782 (0.1313)	0.6479 (0.0636)	0.5906 (0.0596)	0.5122 (0.0607)	0.5122 (0.0536)

Note:

1. Average (se) were calculated for the following model performance: Sensitivity = sensitivity (True Positive Rate) = TP/(TP+FN); Specificity = specificity (True Negative Rate) = TN/(TN+FP); PPV (Positive Predicted Value) = TP/(TP+FP); NPV (Negative Predicted Value) = TN/(TN+FN); Accuracy (ACC) = (TP+TN)/(TP+TN+FP+FN); Balanced Accuracy (BA) = (sensitivity+specificity)/2

2. Standard error (se) was calculated based on Rubin’s rule for imputed data. For the se of AUC in testing data, bootstrapping method repeated 1000 times was used to estimate the within-subject variance.

### Using APAT+EMBARC data for training and external validation

**Fig 5:**
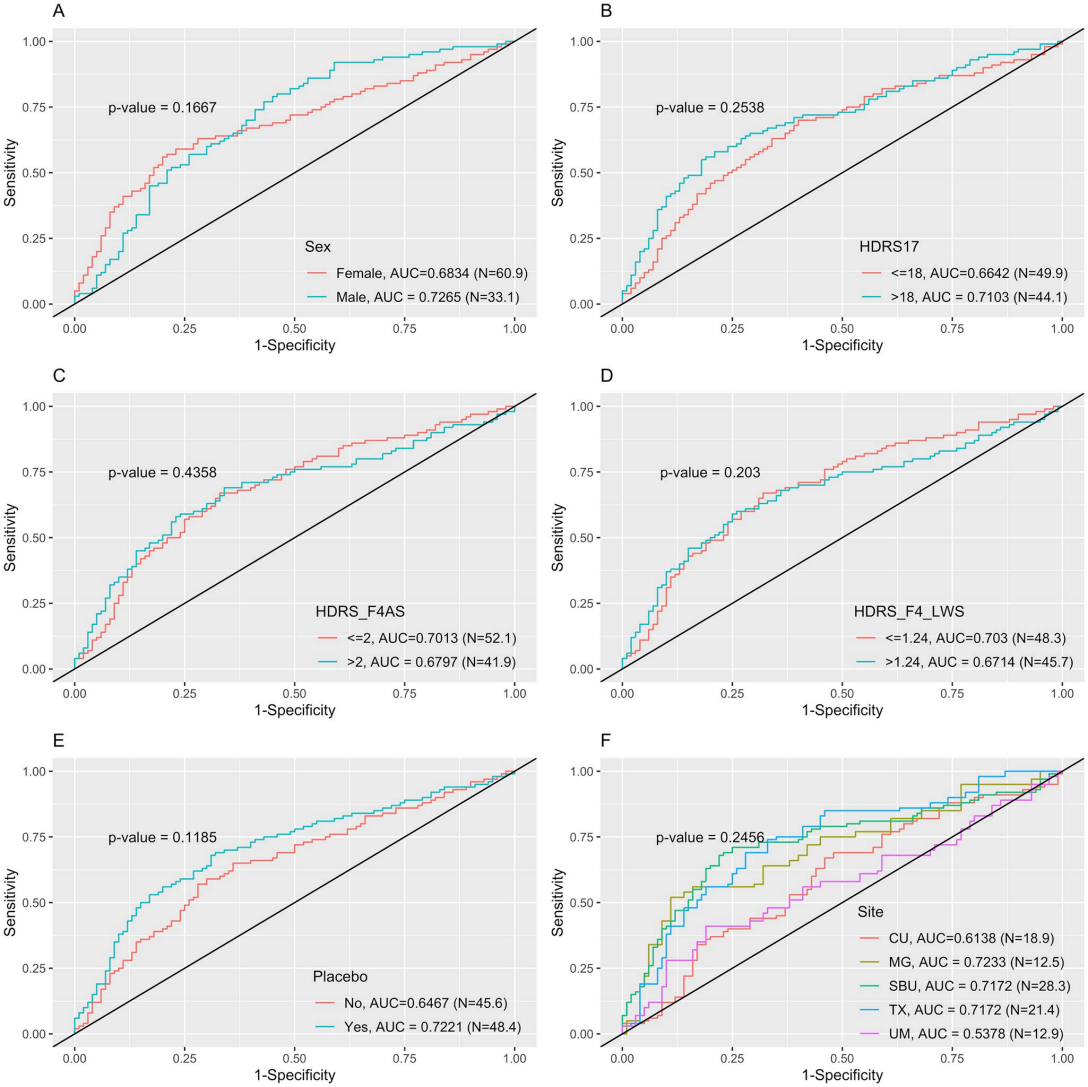
ROC curves comparison across groups based on the stacking model using penalized logistic regression and neural network which had the best predictive performance from internal cross-validation using training set.

The combined APAT+EMBARC data were further divided into 2/3 as training set and 1/3 as testing set to compare the results above. According to the training results in Table 7, the stacking method with results from penalized logistic regression and neural network had the highest accuracy (0.6866 ± 0.0269) and AUC (0.6535 ± 0.0325) values among all 6 models plus 2 stacking methods. Even though the training results from 2/3 combined data were not as good as APAT study, all the model metrics from the testing results were close to the training results, which indicated the validity of our ML pipeline. One of the reasons that caused the lower performances comparing to APAT study such as Accuracy and AUC was that the training data were from 5 different sites and there might existed system bias across different sites causing too much background noises (Fig 5).

**Table 7:** Model performance results based on random forest, gradient boosting, penalized logistic regression, XGBoost, SVM, neural network, and stacking for 2/3 combined data as training set and 1/3 combined data as testing set after multiple imputation for 10 times.

Model	Name	sensitivity (se)	specificity (se)	PPV (se)	NPV (se)	ACC (se)	BA (se)	AUC (se)
Random Forest	Training set 2/3	0.0667 (0.0597)	0.96 (0.0289)	0.4902 (< .0001)	0.6442 (0.0204)	0.6377 (0.0235)	0.5134 (0.0192)	0.5165 (0.021)
	Testing set 1/3	0.0516 (0.0807)	0.9695 (0.0491)	0.4761 (0.2836)	0.6476 (0.082)	0.6394 (0.0779)	0.5106 (0.0549)	0.5106 (0.0475)
Gradient Boosting	Training set 2/3	0.2067 (0.0852)	0.8916 (0.0477)	0.5262 (0.1012)	0.6648 (0.0288)	0.6447 (0.0353)	0.5492 (0.0328)	0.5527 (0.0343)
	Testing set 1/3	0.3277 (0.1706)	0.8368 (0.1054)	0.5271 (0.2303)	0.6934 (0.0919)	0.6543 (0.0675)	0.5823 (0.0636)	0.5822 (0.0577)
SVM	Training set 2/3	0.314 (0.1074)	0.8715 (0.0352)	0.5779 (0.0706)	0.6931 (0.0209)	0.6718 (0.0264)	0.5928 (0.0433)	0.5989 (0.0438)
	Testing set 1/3	0.3687 (0.1169)	0.8474 (0.0944)	0.5888 (0.1779)	0.7066 (0.0766)	0.6755 (0.0697)	0.608 (0.0678)	0.6081 (0.0629)
XGBoost	Training set 2/3	0.3575 (0.0882)	0.7675 (0.0403)	0.4639 (0.0615)	0.679 (0.0253)	0.621 (0.0298)	0.5625 (0.0377)	0.5664 (0.0363)
	Testing set 1/3	0.3961 (0.1046)	0.7901 (0.0815)	0.517 (0.1468)	0.7017 (0.074)	0.65 (0.0592)	0.5931 (0.0582)	0.593 (0.0522)
Penalized logistic regression	Training set 2/3	0.5011 (0.0781)	0.7864 (0.0304)	0.5706 (0.0588)	0.7368 (0.017)	0.6847 (0.0267)	0.6437 (0.0373)	0.6471 (0.0384)
	Testing set 1/3	0.5565 (0.1279)	0.7705 (0.0965)	0.5782 (0.1368)	0.756 (0.0894)	0.6904 (0.0728)	0.6635 (0.0722)	0.6635 (0.0687)
Neural Network	Training set 2/3	0.5199 (0.0481)	0.7801 (0.0248)	0.5736 (0.053)	0.7412 (0.0221)	0.6865 (0.027)	0.65 (0.0308)	0.6532 (0.0327)
	Testing set 1/3	0.5567 (0.144)	0.7409 (0.107)	0.5524 (0.1407)	0.7503 (0.0837)	0.6755 (0.0739)	0.6488 (0.0769)	0.6488 (0.0734)
Stacking (Using above 6 models)	Training set 2/3	0.3384 (0.087)	0.8528 (0.0387)	0.5671 (0.0602)	0.6953 (0.021)	0.6683 (0.027)	0.5956 (0.0354)	0.6007 (0.036)
	Testing set 1/3	0.4123 (0.1322)	0.819 (0.0827)	0.5656 (0.1363)	0.7146 (0.0901)	0.6723 (0.063)	0.6156 (0.0575)	0.6157 (0.0516)
Stacking (Using Penalized logistic regression and Neural Network)	Training set 2/3	0.5205 (0.0481)	0.78 (0.025)	0.5737 (0.0526)	0.7414 (0.0222)	0.6866 (0.0269)	0.6502 (0.0307)	0.6535 (0.0325)
	Testing set 1/3	0.5602 (0.144)	0.7409 (0.1074)	0.5537 (0.1423)	0.7518 (0.0837)	0.6766 (0.075)	0.6506 (0.078)	0.6505 (0.0746)

Note:

1. Average (se) were calculated for the following model performance: Sensitivity = sensitivity (True Positive Rate) = TP/(TP+FN); Specificity = specificity (True Negative Rate) = TN/(TN+FP); PPV (Positive Predicted Value) = TP/(TP+FP); NPV (Negative Predicted Value) = TN/(TN+FN); Accuracy (ACC) = (TP+TN)/(TP+TN+FP+FN); Balanced Accuracy (BA) = (sensitivity+specificity)/2

2. Standard error (se) was calculated based on Rubin’s rule for imputed data. For the se of AUC in testing data, bootstrapping method repeated 1000 times was used to estimate the within-subject variance.

## Discussion

In this paper, we first developed a general machine learning pipeline for prediction of MDD remission after treatment of escitalopram or placebo using 85 participants from APAT data with participants’ clinical and demographic data, data from questionnaires and measurements from MRI brain imaging data. Our proposed ML pipeline included data pre-processing, multiple imputation for missing values, feature selection based on the top 50 most frequent variables, predictive modeling using 6 popular ML models plus 2 stacking methods, and performance evaluation based on repeated cross-validation. Our accuracy reached 83.41%, balance accuracy reached 82.43%, and AUC reached 0.8257, which were much higher than other published papers’ internal validation results. For example, Chekround *et al*. [15] used 1949 patients from STAR*D and their clinical, demographic and psychiatric diagnostic symptom questionnaire to train a predictive model for remission that is defined as a final score of 1-item self-report QIDS < = 5. The AUC value and accuracy of their model are 0.7 and 64.6% from internal cross-validation. However, our ML models did not sustain this performance when externally evaluated, necessitating further investigation.

Some features selected in our model using APAT data were consistent with other studies. For example, Anxiety, which is factor 4 of HDRS, was a top feature with most frequent rank of 8 for arithmetic sum and 11 for load-weighted sum [54] from all our base ML models. It has also been shown to be a top ranked feature by other researchers. Top predictors of Benolt *et al*.’s predictive model included HDRS scale items and anxiety [55]. Both Iniesta *et al*. [56] and Taliaz and his colleagues [16] identified anxiety disorder among their top predictive features. Nunez *et al*. [57] also identified IDAS anxiety score as one of the top predictors. Additionally, Anxiety makes MDD harder to treat, as when the two are presented together, people are more resistant to pharmacological treatment [58–60]. However, the top 50 most frequent features selected by APAT data were much different from that based on 2/3 APAT+EMBARC data or EMBARC data (see Table A2 of S1 File), which might be the reason why the prediction results from using EMBARC data as external validation set were not satisfactory.

When building our ML pipeline, we also tried other possible combinations of each step such as a different number of top ranked features (top 10/15/25/50), different repeated K-fold CV (5 repeated 3-fold, 5 repeated 4-fold, 10 repeated 5-fold), including other ML models such as gradient boosting decision tree, and different number of final selected most frequent features (20/25/30/40). All prediction performances were similar to the results reported here. We further added the early weeks’ (week 1 to week 4) HDRS changing rates from the baseline HDRS scores as additional predictors, and the final prediction results showed slightly but not significantly improvement on the predictive performances.

When using EMBARC data as external validation data, the prediction results for remission were not as good as using the same predictive models generated from APAT data. Overfitting of APAT data could be one of the reasons as this issue may exist in any ML models. In addition, we compared all the features between APAT data and EMBARC data, where almost all the features were significantly different between the two datasets even after adjusting for age, sex, and baseline HDRS 17 (see Table A3 of S1 File). Therefore, the reason why the better results from APAT study could not be generalized to EMBARC study using the same ML pipeline was high likely because of the significant difference between these two datasets. Since the APAT study was from single site but the EMBARC study was from 4 different sites, we further examined the site effects by three steps. First, the ROC curves from EMBARC as testing set were generated by sites, and we noticed the differences among the 4 sites. Second, EMBARC data with a larger sample size were considered as training set and APAT as testing set. There was a significantly drop of model performances when using EMBARC as training set with 4 different sites compared with the single site APAT data. Finally, we used the combined APAT+EMBARC data with 2/3 as training set and 1/3 as testing set. The results from testing set were close to the results from training set, which indicated the validity of our proposed ML pipeline. But all the model performances from training set were worse than those from APAT data, because the 2/3 combined data included 5 sites, which further verified data from multiple sites generally led to worse performances on predicting remission. Several studies also addressed the site differences issue. In 2011, Frank *et al*. [61] evaluated the time to remission in two sites (Pisa and Pittsburgh) and used multiple efforts to minimize the site differences. For example, psychiatrists from Pisa were trained in Pittsburgh for one year to ensure the consistency between sites. However, the time to remission between two types of active treatments was significantly different in Pittsburgh but showed non-significant difference in Pisa. Wu *et al*. [62] questioned whether the high accuracy in predicting remission achieved by their current single site data could be generalized to datasets from multiple sites. Similarly, Xu *et al*. [63] obtained the highest AUC in predicting antidepressant treatment outcome, 0.7654, from gradient boosting decision tree for a single medical center, but lack the generalization to multi-centers because of the geography, insurance, and patients from single site might not fully represent the population at risk for multiple sites. In addition, we examined other effects such as age, sex, HDRS 17, and HDRS factor 4, but none of the ROC curves by these features were significantly different. Therefore, it does not suggest that the predictive performance of the final predictive model was driven by one specific subgroup defined by these features.

### Limitations

One limitation of our study is the small sample size. To address this, we used 5 repeated 4-fold CV for robust evaluation of our predictor’s performance and compared the results from 6 popular ML models plus two stacking methods to ensemble predictions from the base ML models. The fact that our final predictive model had similar or better predictive performance compared with other reported predictive models shows that our advanced ML pipeline may overcome the disadvantage of a small training dataset with sample size <100. The overall ML pipeline we reported here can be used to build a predictive model with any size of training set.

Age is typically found as an important predictor in other studies, however the APAT study consisted of mostly young adults with half of them between the ages of 18–25. This may explain why age is not a top predictor in our list, different from what was found by Kautzky *et al*. [64] and Taliaz *et al*. [16]. However, this is a critical age range for depression studies since this age range is typically when people start to become more independent from parents and the rates of depression start increasing sharply. Additionally, this dataset lacks information or is difficult to assess accurately on other clinical features such as family history or the number of depressive episodes, which may be prognostic factors for remission.

## Supporting information

S1 File This supporting information file contains 5 parts as listed below: A0: Rubin’s rule. Table A1: Data dictionary for questionnaire the Quick Inventory of Depressive Symptomatology (QIDS) and the Hamilton Depression Rating Scale (HDRS). Table A2: Top 50 selected features with rankings from APAT study, EMBARC study, and APAT+EMBARC study when considering each data as training set. Table A3: Comparisons of the features between APAT study and EMBARC study. A4: Example R code for calculating final p-value for comparing different ROC curves and combining ROC curves from imputed datasets.A5: Example Python code for applying our predictive modeling pipeline.
(PDF)
