# Supplementary material for: Examining factors related to low performance of predicting remission in participants with major depressive disorder using neuroimaging data and other clinical features
Source: PLoS One. 2024 Mar 28;19(3):e0299625. doi: 10.1371/journal.pone.0299625 (PMC10977765; doi:10.1371/journal.pone.0299625)
Supplement: S1 File — Table A1: Data dictionary for questionnaire the Quick Inventory of Depressive Symptomatology (QIDS) and the Hamilton Depression Rating Scale (HDRS). Table A2: Top 50 selected features with rankings from APAT study, EMBARC study, and APAT+EMBARC study when considering each data as training set. Table A3: Comparisons of the features between APAT study and EMBARC study. A4: Example R code for calculating final p-value for comparing different ROC curves and combining ROC curves from imputed datasets.A5: Example Python code for applying our predictive modeling pipeline. (PDF) [file pone.0299625.s001.pdf]

Appendix
A0: Rubin's Rule
Table A1: Data dictionary for questionnaire the Quick Inventory of Depressive Symptomatology (QIDS) and the Hamilton Depression Rating Scale (HDRS)
Table A2: Top 50 selected features with rankings from APAT study, EMBARC study, and APAT+EMBARC study when considering each data as training set
Table A3: Comparisons of the features between APAT study and EMBARC study.
A4: Example R code for calculating final p-value for comparing different ROC curves and combining ROC curves from imputed datasets
A5: Example Python code for applying our predictive modeling pipeline

A0: Rubin's Rule (Rubin 2004)**:**
Let $\hat{Q}_i$ and $\hat{U}_i$ denote the point estimate and variance estimate from the ith imputed dataset, $i = 1,2,\ldots,m$. The point estimate Q from multiple imputation is the average of the m imputed datasets:

$$\bar{Q} = \frac{1}{m}\sum_{i=1}^{m}\hat{Q}_i$$

$\bar{U}$ is the within-imputation variance:

$$\bar{U} = \frac{1}{m}\sum_{i=1}^{m}\hat{U}_i$$

B is the between-imputation variance:

$$B = \frac{1}{m-1}\sum_{i=1}^{m}\left(\hat{Q}_i - \bar{Q}\right)^2$$

Then the variance estimate associated with $\bar{Q}$ is the total variance:

$$T = \bar{U} + \left(1 + \frac{1}{m}\right)B$$

Table A1: Data dictionary for questionnaire the Quick Inventory of Depressive Symptomatology (QIDS) and the Hamilton Depression Rating Scale (HDRS).

**Variable Name**	**Label**
**QIDS**	
QIDS_01	1. Falling Asleep:
QIDS_02	2. Sleep During the Night:
QIDS_03	3. Waking up Too Early:
QIDS_04	4. Sleeping Too Much:
QIDS_05	5. Feeling Sad:
QIDS_06	6. Decreased Appetite:
QIDS_07	7. Increased Appetite
QIDS_08	8. Decreased Weight (Within the Last Two Weeks):
QIDS_09	9. Increased Weight (Within the Last Two Weeks):
QIDS_10	10. Concentration/Decision Making:

Variable Name	Label
QIDS_11	11. View of Myself:
QIDS_12	12. Thoughts of Death or Suicide:
QIDS_13	13. General Interest:
QIDS_14	14. Energy Level:
QIDS_15	15. Feeling slowed down:
QIDS_16	16. Feeling Restless:
QIDS_Total	QIDS Total
**HDRS**	
HDRS_1	(H1) DEPRESSED MOOD: (sadness, hopeless, helpless, worthless): What's your mood been like this past week (compared to when you feel OK?) Have been feeling down or depressed? IF YES: Can you describe what this feeling has been like for you? How bad is the feeling? In the last week, how often have you felt (OWN EQUIVALENT FOR DEPRESSED MOOD)? On how many days? For how long each day? How long (MONTHS/YEARS) have you been feeling (OWN WORDS)?
HDRS_23	(H23) HOPELESSNESS: (discouragement, pessimism, hopeless, despair): How are you feeling about the future? If UNKNOWN: Have you been feeling discouraged or pessimistic? IF YES: What have your thoughts been? IF UNKNOWN: How discouraged/pessimistic have you felt? Do you think that you'll get well or that things are going to get better? When people tell you that you will get well or that things will get better, do you feel reassured? In the last week, how often have you felt (OWN EQUIVALENT FOR PESSIMISTIC/DISCOURAGED/HOPELESS)?Every day? All day?
HDRS_7	(H7) WORK AND ACTIVITIES: How have you been spending your time this past week (when not at work)? Have you felt interested in doing (THOSE THINGS), or do you feel you have to push yourself to do them? How much less interested in these things have you been this past week compared to when you're not depressed? How hard do you have to push yourself to do them? Have you stopped doing anything you used to do? IF YES: Why? (What about hobbies?) About how many hours a day do you spend doing things that interest you? Is there anything you look forward to?
HDRS_22	(H22) HELPLESSNESS (lacking confidence in abilities, overwhelmed, needs direction, needs assistance): In the past week, have you been able to get as much done as you usually do (work or chores)? How much less productive or efficient are you compared to before you were depressed? During the past week, did you feel you had trouble coping with routine activities? Were there times when you felt overwhelmed and unable to complete your activities or responsibilities? IF YES: Can you give me an example? How often did you feel this way during the past week? Were these feelings so bad that you would say you felt helpless? Did other people have to encourage or urge you to tend to your work (school) or household responsibilities? During the past week, did you feel that you were giving up trying to cope with life? During the past week, did you need the physical help of others to complete simple activities like grooming, dressing, or eating?
HDRS_4	(H4) INSOMNIA EARLY (Initial Insomnia): Now let's talk about your sleep. What were your usual hours of going to sleep and waking up, before this began? When have you been falling asleep and waking up over the past week? Have you had any trouble falling asleep at the beginning of the night? (Right after you go to bed, how long has it been taking you to fall asleep?) How many nights this week have you had trouble falling asleep? Have you changed the time at which you try to get to sleep since you've been depressed?
HDRS_5	(H5) INSOMNIA MIDDLE: During the past week, have you been waking up in the middle of the night? IF YES: Do you get out of bed? What do you do? (Only to use the bathroom?) When you get back in bed, are you able to fall right back asleep? How long

Variable Name	Label
	does it take you to fall back asleep? Do you wake up more than once during the night? (IF YES: How long does it take for you to fall back asleep each time?) Have you felt your sleeping has been restless or disturbed some nights? How many nights this week have you had that kind of trouble?
HDRS_6	(H6) INSOMNIA LATE (Terminal Insomnia): What time have you been waking up in the morning for the last time, this past week? IF EARLY: Is that with an alarm clock or do you wake up on your own? What time do you usually awake (when you are well)? How many mornings this past week have you awakened early?
HDRS_12	(H12) SOMATIC SYMPTOMS, GASTROINTESTINAL: Decreased Appetite: Now let's talk about your appetite and weight. How has your appetite been this past week? (What about compared to your usual appetite?) IF LESS: How much less than usual? Have you had to force yourself to eat? Have other people had to urge you to eat? (Have you skipped meals?)
HDRS_AI	APPETITE INCREASE: IF MORE: How much more than usual? In the past week, has your appetite been greater than when you feel well or OK? IF YES: Do you want to eat a little more, somewhat more, or much more than when you feel well or OK?
HDRS_IE	INCREASED EATING: above what is required to maintain weight In the past week, have you actually been eating more than when you feel well or OK? IF YES: Do you want to eat a little more, somewhat more, or much more than when you feel well or OK?
HDRS_CC	CARBOHYDRATE CRAVING: above what is required to maintain weight In the last week, have you been craving or eating more starches or sugars? IF YES: Have you been eating or craving starches or sugars more than when you feel well or OK, much more, or has it been irresistible?
HDRS_16	Weight LOSS score
HDRS_13	(H13) SOMATIC SYMPTOMS, GENERAL:
HDRS_14	(H14) GENITAL SYMPTOMS (such as loss of libido, menstrual disturbances): Sometimes, along with depression or anxiety, people might lose interest in sex. This week, how has your interest in sex been? (I'm not asking about actual sexual activity, but about your interest in sex.) Has there been any change in your interest in sex (from when you were feeling OK?) IF YES: How much less interest do you have compared to when you're not depressed? (Is it a little less or a lot less?)
HDRS_2	(H2) FEELINGS OF GUILT: Have you been putting yourself down this past week, feeling you've done things wrong or let others down? IF YES: what have your thoughts been? Have you been feeling guilty about anything that you've done or not done? IF YES: What have your thoughts been? What about things that happened a long time ago? IF UNKNOWN: How often have you thought about that this past week? Have you thought that you've brought (THIS DEPRESSION) on yourself in some way? (Have you been hearing voices or seeing visions in the last week? IF YES: Tell me about them)
HDRS_24	(H24) WORTHLESSNESS: (inadequacy, low self-esteem, inferiority, deluded worthlessness) During the past week, have you felt that you are not as good as other people whom you know and respect? Have you felt that others are better than you? If YES to either of the above: - During the past week, did you feel that you are 'no good' or 'inferior'? - Would you say that you had feelings of being 'worthless'? How often did you feel this way during the past week?
HDRS_3	(H3) SUICIDE: This past week, have you had any thoughts that life is not worth living? What about thinking you would be better off dead? Have you had thoughts of hurting or killing yourself? IF YES: What have you thought about? Have you actually done anything to hurt yourself?
HDRS_10	(H10) ANXIETY, PSYCHIC: Have you been feeling anxious or tense this past week? IF YES: Is this more than normal for you? Have you been feeling irritable this past week? IF YES: Can you give me some examples? How bad has it been? Have you been worrying a lot about little things, things you don't ordinarily worry about? IF YES: Like what, for example? How about worrying about big problems more than you need to? How often

Variable Name	Label
	have you felt this way the past week? Has this caused you any problems or difficulties? IF YES: Like what, for example?
HDRS_11	(H11) ANXIETY, SOMATIC (physiologic concomitants of anxiety, such as GL - dry mouth, gas, indigestion, diarrhea, stomach cramps, belching CV - heart palpitations, headaches Resp - hyperventilating, sighing Urinary frequency Sweating): Tell me if you've had any of the following physical symptoms in the past week. (Read above list) For each SX acknowledged as present: How much has (THE SX) been bothering you this past week? How bad has it gotten? How much of the time, or how often, have you had it? Did (the symptom) interfere at all with your functioning or your usual activities? NOTE: DO NOT RATE SX'S THAT ARE CLEARLY RELATED TO A DOCUMENTED PHYSICAL CONDITION.
HDRS_15	(H15) HYPOCHONDRIASIS: In the last week, how much have your thoughts been focused on your physical health or how your body is feeling (compared to your normal thinking)? (Have you worried a lot about being or becoming physically ill? Have you really been preoccupied with this?) Have you worried a lot that you had a specific medical illness? Do you complain much about how you feel physically? Have you seen a doctor about these problems? IF YES: What did the doctor say?
HDRS_17	(H17) INSIGHT: (Rating based on observation during interview)
HDRS_9	(H9) AGITATION: Have you felt fidgety or restless this last week? Have you found it difficult to stay seated or felt like you need to move around? (Based on observation during interview)
HDRS_8	(H8) RETARDATION (Slowness of thought and speech; impaired concentration; decreased motor activity) Have you felt slowed down in your thinking, speaking, or movement this last week? IF YES: Have others commented on this? (Based on observation during interview)
HDRS_18_DVT	(H18) DIURNAL VARIATION, TYPE Note whether symptoms are regularly (i.e. at least 5 days per week) worse in morning or evening or afternoon. If NO diurnal variation in mood, mark 'none'. Diurnal Variation (Mood Only) During the past week, have you regularly felt better or worse at any particular part of the day, morning, afternoon or evening? IF VARIATION: How much worse do you feel in the (MORNING, AFTERNOON OR EVENING?) IF UNSURE: How much worse do you feel at this time? A little bit worse or a lot worse? When present, mark the severity of the variation in the next question. How many days in the last week did this happen? Additional instructions, from Joseph Trombello at UTSouthwestern(email, 7/6/2016): If a patient denote(s) TWO times of 'typical' variation during the day, (mark) as 'NO VARIATION', as there was not one CONSISTENT time of variation. ... The variation (also) needed to be observed at least 5 out of 7 days, and not attributed to a clear environmental event.
HDRS_18_DVS	(H18) DIURNAL VARIATION, SEVERITYWhen present, mark the severity of the variation: How much worse do you feel at this time? A little bit worse or a lot worse? How many days in the last week did this happen?
HDRS_19	(H19) DEPERSONALIZATION / DEREALIZATION (Such as feelings of unreality and nihilistic ideas) During the past week, have you ever suddenly had the sensation that everything is unreal, or you're in a dream, or cut off from other people in some strange way? Have you felt like you were outside of your body or like you were watching yourself do things? IF YES: Tell me about these feelings. - How bad has that been? - How often this week has that happened? - How many days during the past week did you have these feelings? - Did it interfere with work or home life?
HDRS_20	(H20) PARANOID SYMPTOMS (suspiciousness, persecutory thought, paranoid delusions) This past week, have you thought that anyone was trying to give you a hard time or hurt you? What about talking about you behind your back? IF YES: Tell me about that; - Who and why? - How often?
HDRS_21	(H21) OBSESSIONAL AND COMPULSIVE SYMPTOMS (obsessions, compulsions) In the past week, have there been things you had to do over and over again, like checking

Variable Name	Label
	locks and the doors several times, or washing your hands? Have you had any thoughts that don't make any sense to you, but that keep running over and over in your mind? IF YES TO EITHER: Can you give me examples? IF YES to ANY of the above: How many days this past week did you have these (Repetitive behaviors or disturbing thoughts?) How much of each day? Have these (Repetitive behaviors or disturbing thoughts) interfered with anything?
HDRS_17_Total	HDRS-17 Total:
HDRS_21_Total	HDRS-21 Total:
HDRS_24_Total	HDRS-24 Total:
HDRS_F1_AS	HDRS Factor 1, Arithmetic Sum
HDRS_F2_AS	HDRS Factor 2, Arithmetic Sum
HDRS_F3_AS	HDRS Factor 3, Arithmetic Sum
HDRS_F4_AS	HDRS Factor 4, Arithmetic Sum
HDRS_F5_AS	HDRS Factor 5, Arithmetic Sum
HDRS_F1_LWS	HDRS Factor 1, Load-Weighted Sum
HDRS_F2_LWS	HDRS Factor 2, Load-Weighted Sum
HDRS_F3_LWS	HDRS Factor 3, Load-Weighted Sum
HDRS_F4_LWS	HDRS Factor 4, Load-Weighted Sum
HDRS_F5_LWS	HDRS Factor 5, Load-Weighted Sum

Table A2: Top 50 selected features with rankings from APAT study, EMBARC study, and APAT+EMBARC study when considering each data as training set

Variable	Top 50 variables from 2/3 EMBARC+APAT	Top 50 variables from EMBARC data	Top 50 variables from APAT data
Brain_Stem_Volume	1	1	.
CSF_Volume	2	3	31
QIDS_Q05	3	11	.
_4th_Ventricle_Volume	4	25	18
HDRS_H11	5	29	13
Left_Hippocampus_Volume	6	14	43
HDRS_H14	7	21	.
_3rd_Ventricle_Volume	8	24	26
QIDS_Q16	9	4	.
CC_Posterior_Volume	10	23	33
rh_isthmuscingulate_FoldInd	11	7	.
Left_choroid_plexus_Volume	12	2	.
lh_frontalpole_MeanCurv	13	5	.
CC_Anterior_Volume	14	20	23

Variable	Top 50 variables from 2/3 EMBARC+APAT	Top 50 variables from EMBARC data	Top 50 variables from APAT data
Left_Amygdala_Volume	15	32	14
CC_Mid_Anterior_Volume	16	30	36
Right_Accumbens_area_Volume	17	12	.
Left_Cerebellum_White_Matter_Vol	18	19	39
Left_Pallidum_Volume	19	48	10
CC_Mid_Posterior_Volume	20	31	32
CC_Central_Volume	21	26	28
Left_Cerebellum_Cortex_Volume	22	39	30
Right_Thalamus_Proper_Volume	23	13	37
Left_Lateral_Ventricle_Volume	24	8	42
Left_Accumbens_area_Volume	25	42	29
lh_cuneus_FoldInd	26	.	4
rh_caudalanteriorcingulate_FoldI	27	34	.
Left_Inf_Lat_Vent_Volume	28	22	45
rh_entorhinal_ThickAvg	29	.	.
lh_entorhinal_GrayVol	30	.	.
lh_caudalanteriorcingulate_Thick	31	.	41
lh_caudalanteriorcingulate_GrayV	32	45	.
lh_middletemporal_ThickAvg	33	.	.
Left_Caudate_Volume	34	43	25
rh_parstriangularis_ThickAvg	35	.	.
Left_Putamen_Volume	36	6	17
lh_inferiortemporal_FoldInd	37	.	.
Left_Thalamus_Proper_Volume	38	18	44
rh_fusiform_GrayVol	39	.	6
lh_bankssts_FoldInd	40	49	.
rh_isthmuscingulate_MeanCurv	41	10	.
lh_pericalcarine_ThickAvg	42	.	.
rh_frontalpole_MeanCurv	43	.	.
lh_superiorparietal_FoldInd	44	.	.
Right_Pallidum_Volume	45	.	.
Left_VentralDC_Volume	46	27	.
QIDS_Q14	47	44	.
rh_fusiform_FoldInd	48	38	38

Variable	Top 50 variables from 2/3 EMBARC+APAT	Top 50 variables from EMBARC data	Top 50 variables from APAT data
lh_entorhinal_FoldInd	49	.	5
lh_postcentral_FoldInd	50	.	.
rh_inferiortemporal_GrayVol	.	.	12
lh_paracentral_FoldInd	.	28	.
sex_2	.	46	34
HDRS_F4_AS	.	.	8
totedu	.	.	15
HDRS_H3	.	.	16
lh_inferiorparietal_GrayVol	.	.	48
Right_Amygdala_Volume	.	50	.
rh_inferiortemporal_ThickAvg	.	36	.
lh_lateraloccipital_GrayVol	.	.	3
rh_postcentral_FoldInd	.	15	.
lh_posteriorcingulate_MeanCurv	.	40	.
QIDS_Q15	.	.	9
lh_entorhinal_GrayVol	.	.	1
Right_Putamen_Volume	.	.	19
Brain_Stem_Volume	.	.	24
HDRS_H10	.	.	35
lh_cuneus_GrayVol	.	.	2
Right_choroid_plexus_Volume	.	35	.
lh_frontalpole_FoldInd	.	.	47
lh_parstriangularis_FoldInd	.	9	.
lh_paracentral_MeanCurv	.	.	50
lh_parsorbitalis_GrayVol	.	47	.
lh_paracentral_FoldInd	.	.	21
lh_transversetemporal_MeanCurv	.	.	40
lh_pericalcarine_GrayVol	.	.	7
Right_Lateral_Ventricle_Volume	.	41	.
lh_entorhinal_MeanCurv	.	.	27
rh_temporalpole_ThickAvg	.	.	46
lh_lateraloccipital_ThickAvg	.	.	49
HDRS_F4_LWS	.	.	11
rh_rostralanteriorcingulate_Thic	.	.	20

Variable	Top 50 variables from 2/3 EMBARC+APAT	Top 50 variables from EMBARC data	Top 50 variables from APAT data
lh_lateralorbitofrontal_FoldInd	.	16	.
QIDS_Q01	.	.	22
lh_parahippocampal_MeanCurv	.	17	.
Note: Top 20 ranked variables were output each time from 6 models (Random Forest, Lasso, Ridge, XGBoost, SVM, and Neural Network) with 5-fold cross validation repeated 5 times were iterated over 10 imputed dataset. There were total of 30,000 (20*5*5*6*10) observations. Then, calculate frequency for the above 30,000 variables and keep top 50 most frequent variables for the following modeling.			

Table A3: Comparisons of the features between APAT study and EMBARC study.

Variable	Nmissing	Level	Total	EMBARC	APAT	P-value*	Standardized difference	Estimated difference and 95% CI	P-value**
sex	2	F	180 (64.29%)	124 (63.59%)	56 (65.88%)	0.7850	0.05	-	-
		M	100 (35.71%)	71 (36.41%)	29 (34.12%)			-	
3rd_Ventricle_Volume	0	197 vs 85	783.90±371.60	775.00±378.20	795.30±339.90	0.0832	-0.06	-94.81 (-174.33, -15.29)	0.0196
4th_Ventricle_Volume	0	197 vs 85	1639.35±747.70	1657.00±779.50	1586.80±687.40	0.4365	0.1	72.95 (-82.94, 228.84)	0.3577
Brain_Stem_Volume	0	197 vs 85	20674.40±3196.70	20804.70±3389.70	20500.70±2680.30	0.0842	0.1	657.31 (83.13, 1231.50)	0.0250
CC_Anterior_Volume	0	197 vs 85	830.45±180.10	834.90±175.20	817.10±200.30	0.0498	0.09	40.80 (4.44, 77.17)	0.0280
CC_Central_Volume	0	197 vs 85	461.15±123.00	461.90±115.10	454.90±131.80	0.9803	0.06	3.76 (-21.61, 29.13)	0.7706
CC_Mid_Anterior_Volume	0	197 vs 85	454.85±122.40	452.40±130.20	464.90±102.60	0.9778	-0.11	11.98 (-16.79, 40.76)	0.4130
CC_Mid_Posterior_Volume	0	197 vs 85	439.55±114.80	434.30±113.40	448.40±115.50	0.1555	-0.12	-7.37 (-28.71, 13.98)	0.4975
CC_Posterior_Volume	0	197 vs 85	927.90±166.10	944.40±162.40	892.90±185.30	0.0263	0.3	29.45 (-5.91, 64.80)	0.1022
CSF_Volume	0	197 vs 85	1045.95±323.10	1049.00±390.00	1040.50±229.30	0.8767	0.03	-3.53 (-73.12, 66.06)	0.9205
Left_Accumbens_area_Volume	0	197 vs 85	536.75±190.60	518.00±204.70	587.50±117.70	0.0015	-0.42	-28.83 (-61.17, 3.52)	0.0805
Left_Amygdala_Volume	0	197 vs 85	1553.45±311.70	1557.90±329.90	1552.80±287.60	0.3707	0.02	-10.87 (-67.53, 45.78)	0.7058

Variable	Nmissing	Level	Total	EMBARC	APAT	P-value*	Standardized difference	Estimated difference and 95% CI	P-value**
Left_Caudate_Volume	0	197 vs 85	3621.55±709.80	3605.50±717.40	3645.00±636.00	0.0500	-0.06	-81.52 (-211.72, 48.68)	0.2188
Left_Cerebellum_Cortex_Volume	0	197 vs 85	50833.25±7854.20	51098.50±8199.40	50102.90±6324.70	0.1368	0.14	2156.19 (786.25, 3526.13)	0.0021
Left_Cerebellum_White_Matter_Volume	0	197 vs 85	15487.10±2594.80	15514.80±2813.80	15410.20±2610.80	0.5781	0.04	222.77 (-346.79, 792.32)	0.4420
Left_Hippocampus_Volume	0	197 vs 85	4262.40±580.80	4282.40±572.10	4182.80±578.40	0.2393	0.17	92.79 (-13.67, 199.25)	0.0873
Left_Inf_Lat_Vent_Volume	0	197 vs 85	208.35±152.20	205.50±161.20	209.80±133.60	0.7394	-0.03	-18.83 (-58.45, 20.80)	0.3504
Left_Lateral_Ventricle_Volume	0	197 vs 85	5950.85±4709.90	6079.80±4659.40	5602.30±4111.20	0.1767	0.11	321.73 (-735.16, 1378.61)	0.5495
Left_Pallidum_Volume	0	197 vs 85	1429.70±402.60	1349.60±418.90	1618.20±352.50	<.0001	-0.69	-205.81 (-273.63, -137.98)	<.0001
Left_Putamen_Volume	0	197 vs 85	5527.05±1143.80	5348.40±1188.50	5830.00±842.50	<.0001	-0.47	-301.53 (-489.74, -113.32)	0.0018
Left_Thalamus_Proper_Volume	0	197 vs 85	8309.70±1471.70	8556.10±1397.60	7796.90±1090.50	<.0001	0.61	753.79 (500.96, 1006.61)	<.0001
Left_VentralDC_Volume	0	197 vs 85	3890.25±652.00	3885.10±705.90	3909.60±590.40	0.3258	-0.04	-11.45 (-128.15, 105.25)	0.8469
Left_choroid_plexus_Volume	0	197 vs 85	1167.20±416.00	1198.80±436.90	1073.00±315.70	0.0509	0.33	53.28 (-34.04, 140.60)	0.2307
Right_Accumbens_area_Volume	0	197 vs 85	578.10±161.50	560.80±160.30	611.30±142.80	0.0088	-0.33	-13.67 (-40.02, 12.68)	0.3079
Right_Amygdala_Volume	0	197 vs 85	1666.50±290.40	1681.50±311.90	1632.70±309.00	0.1754	0.16	69.65 (11.54, 127.76)	0.0190
Right_Caudate_Volume	0	197 vs 85	3585.15±760.50	3504.40±848.50	3778.20±673.50	0.0007	-0.36	-194.91 (-331.22, -58.60)	0.0052
Right_Cerebellum_Cortex_Volume	0	197 vs 85	51726.65±8644.60	52194.70±8928.90	51106.50±6990.10	0.0509	0.14	2741.40 (1237.32, 4245.47)	0.0004
Right_Cerebellum_White_Matter_Volume	0	197 vs 85	15651.45±3291.80	15842.20±3421.30	15392.90±2363.40	0.0783	0.15	527.68 (-99.71, 1155.06)	0.0989
Right_Hippocampus_Volume	0	197 vs 85	4349.05±634.50	4351.60±627.10	4335.30±624.50	0.7764	0.03	27.20 (-80.61, 135.02)	0.6198
Right_Inf_Lat_Vent_Volume	0	197 vs 85	231.45±205.70	238.10±208.00	218.60±204.10	0.5270	0.09	-7.27 (-53.86, 39.31)	0.7589

Variable	Nmissing	Level	Total	EMBARC	APAT	P-value*	Standardized difference	Estimated difference and 95% CI	P-value**
Right_Lateral_Ventricle_Volume	0	197 vs 85	5570.25±3974.20	5654.60±3938.70	4887.40±4069.90	0.3238	0.19	112.07 (-819.67, 1043.80)	0.8130
Right_Pallidum_Volume	0	197 vs 85	1475.65±295.30	1452.50±320.00	1514.70±217.80	0.0155	-0.23	-37.67 (-100.06, 24.73)	0.2357
Right_Putamen_Volume	0	197 vs 85	5272.15±1134.70	5152.00±989.30	5596.40±961.80	0.0003	-0.46	-150.76 (-324.93, 23.42)	0.0895
Right_Thalamus_Proper_Volume	0	197 vs 85	7351.65±1148.50	7372.30±1269.40	7312.40±1018.00	0.5657	0.05	79.14 (-138.32, 296.60)	0.4743
Right_VentralDC_Volume	0	197 vs 85	3833.50±622.50	3807.50±653.90	3906.90±528.10	0.0869	-0.17	-26.35 (-146.64, 93.93)	0.6666
Right_choroid_plexus_Volume	0	197 vs 85	1315.30±525.10	1358.00±549.10	1207.80±414.10	0.1052	0.31	35.28 (-71.72, 142.28)	0.5168
lh_bankssts_FoldInd	0	197 vs 85	15.00±7.00	13.00±7.00	18.00±6.00	<.0001	-0.77	-0.46 (-13.57, 12.66)	0.9455
lh_bankssts_GrayVol	0	197 vs 85	2531.00±543.00	2468.00±553.00	2593.00±538.00	0.0898	-0.23	-5.95 (-112.97, 101.07)	0.9129
lh_bankssts_MeanCurv	0	197 vs 85	0.13±0.02	0.12±0.02	0.14±0.02	<.0001	-1	-0.02 (-0.02, -0.01)	<.0001
lh_bankssts_ThickAvg	0	197 vs 85	2.53±0.22	2.52±0.24	2.54±0.20	0.1213	-0.09	-0.01 (-0.05, 0.04)	0.7943
lh_caudalanteriorcingulate_FoldInd	0	197 vs 85	18.00±10.00	17.00±9.00	20.00±8.00	<.0001	-0.35	3.14 (-28.82, 35.10)	0.8468
lh_caudalanteriorcingulate_GrayVol	0	197 vs 85	1698.00±665.00	1670.00±662.00	1745.00±672.00	0.0867	-0.11	-56.44 (-182.72, 69.85)	0.3798
lh_caudalanteriorcingulate_MeanCurv	0	197 vs 85	0.15±0.03	0.14±0.02	0.16±0.02	<.0001	-1	-0.02 (-0.02, -0.02)	<.0001
lh_caudalanteriorcingulate_ThickAvg	0	197 vs 85	2.62±0.33	2.59±0.33	2.70±0.28	0.0004	-0.36	-0.09 (-0.15, -0.02)	0.0070
lh_caudalmiddlefrontal_FoldInd	0	197 vs 85	45.00±23.00	41.00±16.00	61.00±26.00	<.0001	-0.93	-38.67 (-56.21, -21.13)	<.0001
lh_caudalmiddlefrontal_GrayVol	0	197 vs 85	6410.00±1400.00	6374.00±1414.00	6462.00±1402.00	0.4863	-0.06	19.26 (-263.46, 301.97)	0.8934
lh_caudalmiddlefrontal_MeanCurv	0	197 vs 85	0.14±0.02	0.13±0.02	0.15±0.01	<.0001	-1.26	-0.03 (-0.03, -0.02)	<.0001
lh_caudalmiddlefrontal_ThickAvg	0	197 vs 85	2.56±0.21	2.55±0.21	2.59±0.22	0.1640	-0.19	0.00 (-0.04, 0.04)	0.8533
lh_cuneus_FoldInd	0	197 vs 85	46.00±20.00	42.00±13.00	59.00±37.00	<.0001	-0.61	-32.45 (-43.21, -21.68)	<.0001

Variable	Nmissing	Level	Total	EMBARC	APAT	P-value*	Standardized difference	Estimated difference and 95% CI	P-value**
lh_cuneus_GrayVol	0	197 vs 85	2768.00±676.00	2685.00±687.00	2918.00±548.00	0.0006	-0.37	-179.06 (-296.80, -61.32)	0.0030
lh_cuneus_MeanCurv	0	197 vs 85	0.16±0.02	0.16±0.02	0.17±0.01	<.0001	-0.63	-0.01 (-0.01, -0.01)	<.0001
lh_cuneus_ThickAvg	0	197 vs 85	1.82±0.21	1.77±0.22	1.90±0.18	<.0001	-0.65	-0.12 (-0.16, -0.08)	<.0001
lh_entorhinal_FoldInd	0	197 vs 85	9.00±10.00	8.00±7.00	15.00±19.00	<.0001	-0.49	-11.70 (-42.76, 19.37)	0.4593
lh_entorhinal_GrayVol	0	197 vs 85	1764.50±442.00	1834.00±445.00	1620.00±386.00	<.0001	0.51	209.53 (122.77, 296.29)	<.0001
lh_entorhinal_MeanCurv	0	197 vs 85	0.15±0.03	0.14±0.02	0.18±0.03	<.0001	-1.57	-0.04 (-0.05, -0.04)	<.0001
lh_entorhinal_ThickAvg	0	197 vs 85	3.33±0.47	3.38±0.46	3.20±0.39	<.0001	0.42	0.22 (0.13, 0.30)	<.0001
lh_frontalpole_FoldInd	0	197 vs 85	11.00±6.00	10.00±5.00	13.00±5.00	<.0001	-0.6	-4.78 (-7.19, -2.38)	0.0001
lh_frontalpole_GrayVol	0	197 vs 85	788.50±207.00	769.00±191.00	848.00±211.00	0.0032	-0.39	-28.35 (-69.45, 12.75)	0.1756
lh_frontalpole_MeanCurv	0	197 vs 85	0.21±0.03	0.20±0.03	0.22±0.03	<.0001	-0.67	-0.02 (-0.03, -0.01)	<.0001
lh_frontalpole_ThickAvg	0	197 vs 85	2.81±0.38	2.81±0.39	2.84±0.38	0.8517	-0.08	0.03 (-0.04, 0.11)	0.3975
lh_fusiform_FoldInd	0	197 vs 85	97.00±50.00	87.00±32.00	130.00±70.00	<.0001	-0.79	-36.91 (-89.77, 15.95)	0.1703
lh_fusiform_GrayVol	0	197 vs 85	9793.00±2082.00	9814.00±2111.00	9706.00±2050.00	0.7167	0.05	369.44 (11.03, 727.85)	0.0434
lh_fusiform_MeanCurv	0	197 vs 85	0.15±0.02	0.15±0.01	0.18±0.01	<.0001	-3	-0.03 (-0.03, -0.02)	<.0001
lh_fusiform_ThickAvg	0	197 vs 85	2.73±0.22	2.75±0.22	2.68±0.23	0.0311	0.31	0.08 (0.04, 0.12)	0.0001
lh_inferiorparietal_FoldInd	0	197 vs 85	111.00±43.00	104.00±34.00	130.00±42.00	<.0001	-0.68	-32.25 (-61.40, -3.11)	0.0302
lh_inferiorparietal_GrayVol	0	197 vs 85	12083.00±2640.00	12016.00±2570.00	12152.00±2667.00	0.1565	-0.05	-50.92 (-504.09, 402.25)	0.8251
lh_inferiorparietal_MeanCurv	0	197 vs 85	0.14±0.02	0.14±0.01	0.15±0.01	<.0001	-1	-0.02 (-0.02, -0.01)	<.0001
lh_inferiorparietal_ThickAvg	0	197 vs 85	2.47±0.18	2.46±0.18	2.49±0.21	0.0187	-0.15	-0.02 (-0.06, 0.01)	0.2006

Variable	Nmissing	Level	Total	EMBARC	APAT	P-value*	Standardized difference	Estimated difference and 95% CI	P-value**
lh_inferiortemporal_FoldInd	0	197 vs 85	111.50±59.00	97.00±38.00	141.00±89.00	<.0001	-0.64	-81.39 (-283.14, 120.37)	0.4278
lh_inferiortemporal_GrayVol	0	197 vs 85	10318.50±2195.00	10340.00±2216.00	10165.00±1932.00	0.7515	0.08	418.42 (24.47, 812.37)	0.0375
lh_inferiortemporal_MeanCurv	0	197 vs 85	0.15±0.02	0.15±0.01	0.18±0.01	<.0001	-3	-0.03 (-0.03, -0.02)	<.0001
lh_inferiortemporal_ThickAvg	0	197 vs 85	2.79±0.25	2.82±0.24	2.74±0.27	0.0041	0.31	0.09 (0.04, 0.13)	0.0001
lh_insula_FoldInd	0	197 vs 85	77.00±83.00	63.00±50.00	133.00±141.00	<.0001	-0.66	-668.03 (-1237.72, -98.34)	0.0217
lh_insula_GrayVol	0	197 vs 85	6453.50±1216.00	6403.00±1002.00	6672.00±1184.00	0.0261	-0.25	-210.57 (-390.62, -30.52)	0.0221
lh_insula_MeanCurv	0	197 vs 85	0.14±0.02	0.13±0.01	0.16±0.02	<.0001	-1.9	-0.03 (-0.03, -0.02)	<.0001
lh_insula_ThickAvg	0	197 vs 85	3.05±0.22	3.05±0.25	3.05±0.17	0.3771	0	0.00 (-0.04, 0.04)	0.9385
lh_isthmuscingulate_FoldInd	0	197 vs 85	34.00±23.00	31.00±14.00	50.00±47.00	<.0001	-0.55	19.53 (-183.09, 222.15)	0.8497
lh_isthmuscingulate_GrayVol	0	197 vs 85	2513.50±634.00	2478.00±656.00	2647.00±605.00	0.0152	-0.27	-106.40 (-222.93, 10.13)	0.0734
lh_isthmuscingulate_MeanCurv	0	197 vs 85	0.16±0.02	0.15±0.01	0.17±0.01	<.0001	-2	-0.02 (-0.02, -0.02)	<.0001
lh_isthmuscingulate_ThickAvg	0	197 vs 85	2.51±0.31	2.50±0.34	2.54±0.25	0.2349	-0.13	0.01 (-0.05, 0.07)	0.8003
lh_lateraloccipital_FoldInd	0	197 vs 85	137.50±54.00	128.00±43.00	157.00±51.00	<.0001	-0.61	-38.22 (-66.12, -10.33)	0.0074
lh_lateraloccipital_GrayVol	0	197 vs 85	11104.00±1910.00	10893.00±2114.00	11181.00±1600.00	0.0549	-0.15	-233.52 (-597.28, 130.23)	0.2074
lh_lateraloccipital_MeanCurv	0	197 vs 85	0.15±0.01	0.15±0.01	0.16±0.01	<.0001	-1	-0.01 (-0.02, -0.01)	<.0001
lh_lateraloccipital_ThickAvg	0	197 vs 85	2.18±0.17	2.16±0.19	2.22±0.16	<.0001	-0.34	-0.06 (-0.10, -0.03)	0.0003
lh_lateralorbitofrontal_FoldInd	0	197 vs 85	91.00±49.00	79.00±36.00	124.00±59.00	<.0001	-0.92	-129.46 (-254.28, -4.64)	0.0421
lh_lateralorbitofrontal_GrayVol	0	197 vs 85	7435.50±1139.00	7263.00±1256.00	7768.00±1044.00	0.0006	-0.44	-266.86 (-488.29, -45.43)	0.0184
lh_lateralorbitofrontal_MeanCurv	0	197 vs 85	0.16±0.02	0.16±0.01	0.18±0.01	<.0001	-2	-0.03 (-0.03, -0.02)	<.0001

Variable	Nmissing	Level	Total	EMBARC	APAT	P-value*	Standardized difference	Estimated difference and 95% CI	P-value**
lh_lateralorbitofrontal_ThickAvg	0	197 vs 85	2.68±0.24	2.68±0.25	2.67±0.21	0.5619	0.04	0.04 (-0.00, 0.09)	0.0556
lh_lingual_FoldInd	0	197 vs 85	96.50±54.00	87.00±35.00	128.00±45.00	<.0001	-1.02	-42.15 (-57.09, -27.21)	<.0001
lh_lingual_GrayVol	0	197 vs 85	6342.50±1441.00	6207.00±1458.00	6531.00±1205.00	0.0201	-0.24	-225.32 (-495.10, 44.46)	0.1013
lh_lingual_MeanCurv	0	197 vs 85	0.16±0.02	0.16±0.01	0.18±0.01	<.0001	-2	-0.02 (-0.02, -0.01)	<.0001
lh_lingual_ThickAvg	0	197 vs 85	1.99±0.19	1.96±0.21	2.03±0.15	0.0001	-0.38	-0.05 (-0.09, -0.01)	0.0067
lh_medialorbitofrontal_FoldInd	0	197 vs 85	84.00±57.00	74.00±52.00	94.00±73.00	0.0003	-0.32	5.86 (-47.63, 59.34)	0.8295
lh_medialorbitofrontal_GrayVol	0	197 vs 85	4865.50±979.00	4869.00±964.00	4865.00±965.00	0.9803	0	91.93 (-82.26, 266.13)	0.2997
lh_medialorbitofrontal_MeanCurv	0	197 vs 85	0.16±0.02	0.16±0.02	0.17±0.02	<.0001	-0.5	-0.02 (-0.02, -0.01)	<.0001
lh_medialorbitofrontal_ThickAvg	0	197 vs 85	2.44±0.22	2.44±0.23	2.44±0.18	0.6943	0	0.03 (-0.02, 0.07)	0.2255
lh_middletemporal_FoldInd	0	197 vs 85	90.00±46.00	80.00±32.00	116.00±50.00	<.0001	-0.86	-60.73 (-90.71, -30.76)	<.0001
lh_middletemporal_GrayVol	0	197 vs 85	10424.50±2221.00	10343.00±2237.00	10671.00±2362.00	0.0290	-0.14	-187.47 (-560.76, 185.82)	0.3237
lh_middletemporal_MeanCurv	0	197 vs 85	0.15±0.02	0.15±0.01	0.17±0.01	<.0001	-2	-0.02 (-0.03, -0.02)	<.0001
lh_middletemporal_ThickAvg	0	197 vs 85	2.91±0.24	2.90±0.24	2.94±0.27	0.2136	-0.16	0.01 (-0.03, 0.05)	0.7142
lh_paracentral_FoldInd	0	197 vs 85	36.00±42.00	27.00±26.00	67.00±79.00	<.0001	-0.68	-754.75 (-1533.54, 24.04)	0.0575
lh_paracentral_GrayVol	0	197 vs 85	3331.00±681.00	3315.00±633.00	3388.00±841.00	0.0541	-0.1	-68.84 (-194.30, 56.63)	0.2811
lh_paracentral_MeanCurv	0	197 vs 85	0.13±0.03	0.13±0.02	0.15±0.02	<.0001	-1	-0.03 (-0.03, -0.02)	<.0001
lh_paracentral_ThickAvg	0	197 vs 85	2.36±0.21	2.33±0.21	2.40±0.20	<.0001	-0.34	-0.06 (-0.10, -0.03)	0.0014
lh_parahippocampal_FoldInd	0	197 vs 85	14.00±11.00	11.00±8.00	21.00±14.00	<.0001	-0.88	-22.16 (-36.25, -8.07)	0.0022
lh_parahippocampal_GrayVol	0	197 vs 85	2242.50±445.00	2254.00±445.00	2194.00±463.00	0.6229	0.13	57.35 (-22.48, 137.19)	0.1584

Variable	Nmissing	Level	Total	EMBARC	APAT	P-value*	Standardized difference	Estimated difference and 95% CI	P-value**
lh_parahippocampal_MeanCurv	0	197 vs 85	0.13±0.04	0.12±0.02	0.16±0.03	<.0001	-1.57	-0.04 (-0.04, -0.03)	<.0001
lh_parahippocampal_ThickAvg	0	197 vs 85	2.79±0.47	2.79±0.49	2.75±0.39	0.5034	0.09	0.03 (-0.05, 0.12)	0.4387
lh_parsopercularis_FoldInd	0	197 vs 85	37.00±16.00	33.00±14.00	45.00±19.00	<.0001	-0.72	-22.42 (-42.92, -1.93)	0.0321
lh_parsopercularis_GrayVol	0	197 vs 85	4741.50±1179.00	4671.00±1359.00	4837.00±820.00	0.0943	-0.15	-53.16 (-257.98, 151.66)	0.6098
lh_parsopercularis_MeanCurv	0	197 vs 85	0.14±0.02	0.13±0.01	0.16±0.01	<.0001	-3	-0.02 (-0.03, -0.02)	<.0001
lh_parsopercularis_ThickAvg	0	197 vs 85	2.61±0.22	2.57±0.22	2.65±0.22	0.0031	-0.36	-0.03 (-0.07, 0.01)	0.1349
lh_parsorbitalis_FoldInd	0	197 vs 85	21.00±11.00	19.00±8.00	27.00±11.00	<.0001	-0.83	-5.07 (-26.41, 16.27)	0.6401
lh_parsorbitalis_GrayVol	0	197 vs 85	2143.00±460.00	2087.00±436.00	2257.00±528.00	0.0031	-0.35	-76.61 (-151.19, -2.03)	0.0441
lh_parsorbitalis_MeanCurv	0	197 vs 85	0.17±0.03	0.16±0.02	0.18±0.02	<.0001	-1	-0.02 (-0.03, -0.02)	<.0001
lh_parsorbitalis_ThickAvg	0	197 vs 85	2.78±0.32	2.78±0.33	2.78±0.24	0.8380	0	0.05 (-0.01, 0.10)	0.1294
lh_parstriangularis_FoldInd	0	197 vs 85	29.50±12.00	28.00±10.00	37.00±13.00	<.0001	-0.78	-8.40 (-12.00, -4.80)	<.0001
lh_parstriangularis_GrayVol	0	197 vs 85	3642.00±828.00	3532.00±817.00	3731.00±609.00	0.0052	-0.28	-118.24 (-266.00, 29.51)	0.1163
lh_parstriangularis_MeanCurv	0	197 vs 85	0.14±0.02	0.14±0.01	0.16±0.02	<.0001	-1.26	-0.02 (-0.02, -0.02)	<.0001
lh_parstriangularis_ThickAvg	0	197 vs 85	2.47±0.21	2.45±0.21	2.53±0.21	0.0675	-0.38	0.00 (-0.04, 0.05)	0.8273
lh_pericalcarine_FoldInd	0	197 vs 85	35.00±22.00	33.00±15.00	45.00±26.00	<.0001	-0.57	-6.31 (-25.51, 12.89)	0.5183
lh_pericalcarine_GrayVol	0	197 vs 85	1907.50±555.00	1817.00±552.00	2057.00±452.00	<.0001	-0.48	-204.16 (-305.17, -103.16)	<.0001
lh_pericalcarine_MeanCurv	0	197 vs 85	0.16±0.02	0.15±0.02	0.17±0.02	<.0001	-1	-0.01 (-0.02, -0.01)	<.0001
lh_pericalcarine_ThickAvg	0	197 vs 85	1.58±0.25	1.50±0.25	1.67±0.19	<.0001	-0.77	-0.16 (-0.20, -0.11)	<.0001
lh_postcentral_FoldInd	0	197 vs 85	107.00±67.00	93.00±42.00	141.00±76.00	<.0001	-0.78	-62.26 (-121.07, -3.44)	0.0381

Variable	Nmissing	Level	Total	EMBARC	APAT	P-value*	Standardized difference	Estimated difference and 95% CI	P-value**
lh_postcentral_GrayVol	0	197 vs 85	9398.00±1883.00	9234.00±1852.00	9655.00±1609.00	0.0016	-0.24	-422.12 (-743.68, -100.57)	0.0103
lh_postcentral_MeanCurv	0	197 vs 85	0.14±0.03	0.13±0.02	0.16±0.01	<.0001	-1.9	-0.03 (-0.03, -0.02)	<.0001
lh_postcentral_ThickAvg	0	197 vs 85	2.08±0.16	2.06±0.16	2.15±0.16	<.0001	-0.56	-0.10 (-0.13, -0.06)	<.0001
lh_posteriorcingulate_FoldInd	0	197 vs 85	37.50±17.00	34.00±13.00	47.00±21.00	<.0001	-0.74	-27.60 (-37.61, -17.59)	<.0001
lh_posteriorcingulate_GrayVol	0	197 vs 85	3048.00±690.00	2982.00±639.00	3276.00±639.00	0.0087	-0.46	-86.07 (-204.38, 32.24)	0.1532
lh_posteriorcingulate_MeanCurv	0	197 vs 85	0.16±0.02	0.16±0.01	0.17±0.01	<.0001	-1	-0.02 (-0.02, -0.01)	<.0001
lh_posteriorcingulate_ThickAvg	0	197 vs 85	2.54±0.22	2.52±0.21	2.61±0.22	0.0021	-0.42	-0.03 (-0.07, 0.01)	0.1252
lh_precentral_FoldInd	0	197 vs 85	163.50±179.00	122.00±116.00	279.00±364.00	<.0001	-0.58	-160.43 (-411.09, 90.23)	0.2087
lh_precentral_GrayVol	0	197 vs 85	12973.50±2272.00	12859.00±2329.00	13311.00±1948.00	0.0735	-0.21	-105.86 (-472.20, 260.47)	0.5699
lh_precentral_MeanCurv	0	197 vs 85	0.14±0.03	0.13±0.02	0.16±0.02	<.0001	-1.5	-0.03 (-0.04, -0.03)	<.0001
lh_precentral_ThickAvg	0	197 vs 85	2.54±0.20	2.53±0.19	2.56±0.18	0.0433	-0.16	-0.02 (-0.05, 0.02)	0.3364
lh_precuneus_FoldInd	0	197 vs 85	94.00±39.00	85.00±28.00	117.00±41.00	<.0001	-0.91	-28.49 (-74.55, 17.57)	0.2244
lh_precuneus_GrayVol	0	197 vs 85	9310.50±1708.00	9244.00±1878.00	9549.00±1388.00	0.0670	-0.18	-61.65 (-365.91, 242.61)	0.6903
lh_precuneus_MeanCurv	0	197 vs 85	0.15±0.01	0.14±0.01	0.16±0.01	<.0001	-2	-0.02 (-0.02, -0.01)	<.0001
lh_precuneus_ThickAvg	0	197 vs 85	2.37±0.21	2.36±0.21	2.41±0.20	0.0002	-0.24	-0.05 (-0.09, -0.01)	0.0106
lh_rostralanteriorcingulate_FoldInd	0	197 vs 85	30.00±14.00	27.00±12.00	37.00±15.00	<.0001	-0.74	-27.26 (-53.62, -0.90)	0.0427
lh_rostralanteriorcingulate_GrayVol	0	197 vs 85	2626.00±671.00	2580.00±655.00	2729.00±595.00	0.0395	-0.24	-76.29 (-210.27, 57.68)	0.2632
lh_rostralanteriorcingulate_MeanCurv	0	197 vs 85	0.16±0.03	0.16±0.02	0.18±0.02	<.0001	-1	-0.02 (-0.03, -0.02)	<.0001
lh_rostralanteriorcingulate_ThickAvg	0	197 vs 85	2.88±0.30	2.85±0.30	2.92±0.26	0.0223	-0.25	-0.03 (-0.08, 0.03)	0.2927

Variable	Nmissing	Level	Total	EMBARC	APAT	P-value*	Standardized difference	Estimated difference and 95% CI	P-value**
lh_rostralmiddlefrontal_FoldInd	0	197 vs 85	180.00±75.00	158.00±52.00	233.00±68.00	<.0001	-1.24	-90.34 (-118.93, -61.75)	<.0001
lh_rostralmiddlefrontal_GrayVol	0	197 vs 85	15204.50±2898.00	15053.00±2925.00	15822.00±2923.00	0.0045	-0.26	-330.75 (-849.20, 187.70)	0.2102
lh_rostralmiddlefrontal_MeanCurv	0	197 vs 85	0.16±0.02	0.15±0.01	0.17±0.01	<.0001	-2	-0.02 (-0.02, -0.02)	<.0001
lh_rostralmiddlefrontal_ThickAvg	0	197 vs 85	2.40±0.20	2.38±0.20	2.44±0.17	0.0106	-0.32	-0.02 (-0.05, 0.02)	0.4029
lh_superiorfrontal_FoldInd	0	197 vs 85	181.50±94.00	159.00±69.00	236.00±92.00	<.0001	-0.95	-134.20 (-209.70, -58.70)	0.0005
lh_superiorfrontal_GrayVol	0	197 vs 85	21805.00±3640.00	21688.00±3413.00	22519.00±3809.00	0.1077	-0.23	-86.09 (-665.98, 493.79)	0.7703
lh_superiorfrontal_MeanCurv	0	197 vs 85	0.15±0.02	0.14±0.01	0.16±0.02	<.0001	-1.26	-0.02 (-0.03, -0.02)	<.0001
lh_superiorfrontal_ThickAvg	0	197 vs 85	2.75±0.24	2.74±0.25	2.79±0.23	0.0208	-0.21	-0.01 (-0.05, 0.03)	0.6628
lh_superiorparietal_FoldInd	0	197 vs 85	126.00±55.00	113.00±41.00	150.00±47.00	<.0001	-0.84	-50.06 (-74.85, -25.27)	<.0001
lh_superiorparietal_GrayVol	0	197 vs 85	12585.50±2048.00	12358.00±2059.00	12992.00±1826.00	0.0035	-0.33	-353.18 (-770.94, 64.59)	0.0972
lh_superiorparietal_MeanCurv	0	197 vs 85	0.14±0.02	0.13±0.01	0.15±0.01	<.0001	-2	-0.02 (-0.02, -0.02)	<.0001
lh_superiorparietal_ThickAvg	0	197 vs 85	2.18±0.18	2.17±0.16	2.23±0.19	<.0001	-0.34	-0.07 (-0.10, -0.04)	<.0001
lh_superiortemporal_FoldInd	0	197 vs 85	81.00±49.00	71.00±35.00	100.00±46.00	<.0001	-0.71	-58.48 (-94.53, -22.43)	0.0016
lh_superiortemporal_GrayVol	0	197 vs 85	11785.50±2085.00	11650.00±2059.00	12350.00±2166.00	0.0054	-0.33	-289.64 (-658.82, 79.54)	0.1236
lh_superiortemporal_MeanCurv	0	197 vs 85	0.13±0.02	0.13±0.01	0.15±0.02	<.0001	-1.26	-0.03 (-0.03, -0.02)	<.0001
lh_superiortemporal_ThickAvg	0	197 vs 85	2.82±0.23	2.80±0.23	2.83±0.21	0.0437	-0.14	-0.01 (-0.05, 0.03)	0.5529
lh_supramarginal_FoldInd	0	197 vs 85	92.00±36.00	86.00±31.00	111.00±38.00	<.0001	-0.72	-42.38 (-63.70, -21.06)	0.0001
lh_supramarginal_GrayVol	0	197 vs 85	10545.00±2192.00	10291.00±2185.00	10788.00±2182.00	0.0933	-0.23	-134.65 (-523.04, 253.73)	0.4955
lh_supramarginal_MeanCurv	0	197 vs 85	0.15±0.02	0.14±0.01	0.16±0.01	<.0001	-2	-0.02 (-0.02, -0.02)	<.0001

Variable	Nmissing	Level	Total	EMBARC	APAT	P-value*	Standardized difference	Estimated difference and 95% CI	P-value**
lh_supramarginal_ThickAvg	0	197 vs 85	2.58±0.21	2.56±0.19	2.61±0.23	0.0144	-0.24	-0.02 (-0.06, 0.01)	0.2514
lh_temporalpole_FoldInd	0	197 vs 85	17.00±12.00	14.00±6.00	26.00±15.00	<.0001	-1.05	-25.39 (-34.21, -16.57)	<.0001
lh_temporalpole_GrayVol	0	197 vs 85	2478.00±582.00	2548.00±566.00	2362.00±516.00	0.0003	0.34	199.63 (96.95, 302.32)	0.0002
lh_temporalpole_MeanCurv	0	197 vs 85	0.18±0.04	0.17±0.03	0.21±0.03	<.0001	-1.33	-0.04 (-0.05, -0.04)	<.0001
lh_temporalpole_ThickAvg	0	197 vs 85	3.67±0.44	3.73±0.39	3.49±0.45	<.0001	0.57	0.23 (0.15, 0.32)	<.0001
lh_transversetemporal_FoldInd	0	197 vs 85	12.00±10.00	10.00±5.00	22.00±21.00	<.0001	-0.79	-25.63 (-32.04, -19.22)	<.0001
lh_transversetemporal_GrayVol	0	197 vs 85	1113.50±322.00	1092.00±289.00	1154.00±322.00	0.1941	-0.2	-2.75 (-54.65, 49.15)	0.9170
lh_transversetemporal_MeanCurv	0	197 vs 85	0.14±0.03	0.14±0.02	0.17±0.02	<.0001	-1.5	-0.04 (-0.04, -0.03)	<.0001
lh_transversetemporal_ThickAvg	0	197 vs 85	2.34±0.30	2.35±0.30	2.32±0.28	0.6456	0.1	0.01 (-0.04, 0.07)	0.6460
rh_bankssts_FoldInd	0	197 vs 85	13.00±6.00	12.00±5.00	16.00±7.00	<.0001	-0.66	-5.70 (-9.45, -1.96)	0.0029
rh_bankssts_GrayVol	0	197 vs 85	2303.00±469.00	2282.00±460.00	2404.00±503.00	0.0734	-0.25	-12.22 (-100.02, 75.58)	0.7843
rh_bankssts_MeanCurv	0	197 vs 85	0.12±0.02	0.12±0.02	0.13±0.02	<.0001	-0.5	-0.01 (-0.02, -0.01)	<.0001
rh_bankssts_ThickAvg	0	197 vs 85	2.60±0.25	2.58±0.25	2.63±0.23	0.0437	-0.21	-0.02 (-0.06, 0.02)	0.3507
rh_caudalanteriorcingulate_FoldInd	0	197 vs 85	22.00±10.00	20.00±8.00	27.00±11.00	<.0001	-0.73	-5.19 (-15.26, 4.88)	0.3113
rh_caudalanteriorcingulate_GrayVol	0	197 vs 85	2143.50±602.00	2129.00±657.00	2165.00±428.00	0.6050	-0.06	21.01 (-105.07, 147.08)	0.7431
rh_caudalanteriorcingulate_MeanCurv	0	197 vs 85	0.15±0.02	0.15±0.02	0.17±0.01	<.0001	-1.26	-0.02 (-0.02, -0.02)	<.0001
rh_caudalanteriorcingulate_ThickAvg	0	197 vs 85	2.54±0.27	2.54±0.28	2.55±0.25	0.6291	-0.04	0.01 (-0.05, 0.06)	0.8267
rh_caudalmiddlefrontal_FoldInd	0	197 vs 85	40.50±22.00	36.00±15.00	54.00±23.00	<.0001	-0.93	0.95 (-74.92, 76.82)	0.9803
rh_caudalmiddlefrontal_GrayVol	0	197 vs 85	5858.00±1620.00	5890.00±1585.00	5793.00±1620.00	0.9378	0.06	114.82 (-164.84, 394.49)	0.4196

Variable	Nmissing	Level	Total	EMBARC	APAT	P-value*	Standardized difference	Estimated difference and 95% CI	P-value**
rh_caudalmiddlefrontal_MeanCurv	0	197 vs 85	0.14±0.02	0.13±0.02	0.15±0.01	<.0001	-1.26	-0.03 (-0.03, -0.02)	<.0001
rh_caudalmiddlefrontal_ThickAvg	0	197 vs 85	2.55±0.21	2.54±0.20	2.56±0.21	0.3389	-0.1	0.01 (-0.03, 0.05)	0.7429
rh_cuneus_FoldInd	0	197 vs 85	49.00±20.00	43.00±15.00	62.00±22.00	<.0001	-1.01	-21.43 (-29.75, -13.11)	<.0001
rh_cuneus_GrayVol	0	197 vs 85	2934.50±619.00	2873.00±573.00	3083.00±600.00	0.0005	-0.36	-186.70 (-312.35, -61.05)	0.0037
rh_cuneus_MeanCurv	0	197 vs 85	0.17±0.02	0.16±0.02	0.18±0.01	<.0001	-1.26	-0.01 (-0.02, -0.01)	<.0001
rh_cuneus_ThickAvg	0	197 vs 85	1.82±0.22	1.79±0.21	1.90±0.22	<.0001	-0.51	-0.11 (-0.15, -0.07)	<.0001
rh_entorhinal_FoldInd	0	197 vs 85	8.00±8.00	7.00±6.00	13.00±10.00	<.0001	-0.73	-21.28 (-36.51, -6.05)	0.0063
rh_entorhinal_GrayVol	0	197 vs 85	1571.50±499.00	1571.00±479.00	1572.00±468.00	0.7305	0	-22.41 (-111.91, 67.10)	0.6225
rh_entorhinal_MeanCurv	0	197 vs 85	0.15±0.04	0.14±0.03	0.18±0.03	<.0001	-1.33	-0.04 (-0.05, -0.04)	<.0001
rh_entorhinal_ThickAvg	0	197 vs 85	3.49±0.57	3.49±0.55	3.50±0.63	0.6536	-0.02	0.01 (-0.10, 0.11)	0.8660
rh_frontalpole_FoldInd	0	197 vs 85	15.50±10.00	14.00±7.00	21.00±10.00	<.0001	-0.81	-18.75 (-32.99, -4.51)	0.0101
rh_frontalpole_GrayVol	0	197 vs 85	1056.00±258.00	1021.00±236.00	1127.00±276.00	0.0008	-0.41	-50.41 (-99.03, -1.78)	0.0422
rh_frontalpole_MeanCurv	0	197 vs 85	0.21±0.03	0.20±0.03	0.23±0.03	<.0001	-1	-0.02 (-0.03, -0.02)	<.0001
rh_frontalpole_ThickAvg	0	197 vs 85	2.76±0.39	2.76±0.43	2.75±0.29	0.5673	0.03	0.01 (-0.07, 0.09)	0.7825
rh_fusiform_FoldInd	0	197 vs 85	92.00±52.00	83.00±31.00	124.00±68.00	<.0001	-0.78	-68.90 (-125.00, -12.81)	0.0162
rh_fusiform_GrayVol	0	197 vs 85	9394.00±2070.00	9373.00±2162.00	9517.00±1719.00	0.4026	-0.07	43.40 (-317.24, 404.04)	0.8129
rh_fusiform_MeanCurv	0	197 vs 85	0.15±0.02	0.15±0.01	0.17±0.01	<.0001	-2	-0.02 (-0.02, -0.02)	<.0001
rh_fusiform_ThickAvg	0	197 vs 85	2.72±0.24	2.73±0.25	2.70±0.23	0.9347	0.12	0.03 (-0.02, 0.07)	0.2534
rh_inferiorparietal_FoldInd	0	197 vs 85	135.00±52.00	123.00±41.00	160.00±51.00	<.0001	-0.8	-42.25 (-58.02, -26.47)	<.0001

Variable	Nmissing	Level	Total	EMBARC	APAT	P-value*	Standardized difference	Estimated difference and 95% CI	P-value**
rh_inferiorparietal_GrayVol	0	197 vs 85	14508.50±3120.00	14185.00±3149.00	15017.00±3208.00	0.0043	-0.26	-534.34 (-1052.32, -16.36)	0.0432
rh_inferiorparietal_MeanCurv	0	197 vs 85	0.14±0.01	0.14±0.01	0.15±0.01	<.0001	-1	-0.01 (-0.01, -0.01)	<.0001
rh_inferiorparietal_ThickAvg	0	197 vs 85	2.49±0.18	2.48±0.17	2.54±0.20	0.0010	-0.32	-0.04 (-0.08, -0.01)	0.0165
rh_inferiortemporal_FoldInd	0	197 vs 85	106.00±54.00	94.00±42.00	136.00±55.00	<.0001	-0.86	-46.80 (-103.46, 9.85)	0.1050
rh_inferiortemporal_GrayVol	0	197 vs 85	10016.00±2322.00	9988.00±2436.00	10089.00±1822.00	0.6861	-0.05	254.68 (-154.44, 663.80)	0.2214
rh_inferiortemporal_MeanCurv	0	197 vs 85	0.15±0.02	0.15±0.01	0.17±0.01	<.0001	-2	-0.02 (-0.02, -0.02)	<.0001
rh_inferiortemporal_ThickAvg	0	197 vs 85	2.80±0.23	2.81±0.23	2.79±0.21	0.2383	0.09	0.06 (0.02, 0.11)	0.0049
rh_insula_FoldInd	0	197 vs 85	92.00±98.00	76.00±75.00	144.00±153.00	<.0001	-0.56	-1350.91 (-2357.88, -343.95)	0.0087
rh_insula_GrayVol	0	197 vs 85	6557.00±1123.00	6509.00±995.00	6849.00±1043.00	0.0035	-0.33	-253.72 (-444.44, -63.00)	0.0093
rh_insula_MeanCurv	0	197 vs 85	0.15±0.02	0.14±0.01	0.17±0.02	<.0001	-1.9	-0.03 (-0.03, -0.02)	<.0001
rh_insula_ThickAvg	0	197 vs 85	3.02±0.25	3.00±0.27	3.07±0.20	0.0055	-0.29	-0.03 (-0.07, 0.01)	0.1569
rh_isthmuscingulate_FoldInd	0	197 vs 85	33.00±23.00	29.00±16.00	43.00±29.00	<.0001	-0.6	-38.51 (-68.91, -8.11)	0.0132
rh_isthmuscingulate_GrayVol	0	197 vs 85	2413.00±545.00	2378.00±552.00	2482.00±548.00	0.1443	-0.19	-30.14 (-140.45, 80.18)	0.5911
rh_isthmuscingulate_MeanCurv	0	197 vs 85	0.16±0.02	0.15±0.02	0.17±0.01	<.0001	-1.26	-0.02 (-0.02, -0.02)	<.0001
rh_isthmuscingulate_ThickAvg	0	197 vs 85	2.46±0.28	2.46±0.28	2.47±0.28	0.3902	-0.04	0.00 (-0.05, 0.05)	0.9435
rh_lateraloccipital_FoldInd	0	197 vs 85	133.00±56.00	121.00±34.00	163.00±71.00	<.0001	-0.75	-37.22 (-84.10, 9.65)	0.1191
rh_lateraloccipital_GrayVol	0	197 vs 85	11003.00±2132.00	10918.00±2272.00	11241.00±1735.00	0.0435	-0.16	-237.54 (-650.44, 175.36)	0.2584
rh_lateraloccipital_MeanCurv	0	197 vs 85	0.16±0.01	0.15±0.01	0.16±0.01	<.0001	-1	-0.01 (-0.02, -0.01)	<.0001
rh_lateraloccipital_ThickAvg	0	197 vs 85	2.23±0.19	2.20±0.19	2.29±0.19	<.0001	-0.47	-0.08 (-0.12, -0.05)	<.0001

Variable	Nmissing	Level	Total	EMBARC	APAT	P-value*	Standardized difference	Estimated difference and 95% CI	P-value**
rh_lateralorbitofrontal_FoldInd	0	197 vs 85	99.00±68.00	88.00±46.00	122.00±72.00	<.0001	-0.56	-23.27 (-57.15, 10.61)	0.1774
rh_lateralorbitofrontal_GrayVol	0	197 vs 85	7140.50±1097.00	7154.00±1205.00	7136.00±851.00	0.8479	0.02	213.83 (-2.30, 429.96)	0.0525
rh_lateralorbitofrontal_MeanCurv	0	197 vs 85	0.16±0.02	0.16±0.01	0.18±0.02	<.0001	-1.26	-0.03 (-0.03, -0.02)	<.0001
rh_lateralorbitofrontal_ThickAvg	0	197 vs 85	2.62±0.25	2.63±0.27	2.59±0.23	0.0080	0.16	0.09 (0.04, 0.13)	0.0001
rh_lingual_FoldInd	0	197 vs 85	101.00±57.00	89.00±35.00	144.00±67.00	<.0001	-1.03	-91.86 (-126.16, -57.57)	<.0001
rh_lingual_GrayVol	0	197 vs 85	6580.00±1315.00	6480.00±1428.00	6763.00±1165.00	0.1061	-0.22	-56.68 (-315.22, 201.86)	0.6664
rh_lingual_MeanCurv	0	197 vs 85	0.17±0.02	0.16±0.01	0.18±0.01	<.0001	-2	-0.02 (-0.02, -0.02)	<.0001
rh_lingual_ThickAvg	0	197 vs 85	2.04±0.21	2.02±0.22	2.07±0.17	0.0011	-0.25	-0.04 (-0.08, -0.01)	0.0234
rh_medialorbitofrontal_FoldInd	0	197 vs 85	75.00±47.00	69.00±34.00	97.00±54.00	<.0001	-0.62	-16.97 (-36.18, 2.24)	0.0832
rh_medialorbitofrontal_GrayVol	0	197 vs 85	4908.00±914.00	4893.00±871.00	4968.00±957.00	0.2099	-0.08	-33.74 (-196.11, 128.64)	0.6828
rh_medialorbitofrontal_MeanCurv	0	197 vs 85	0.16±0.02	0.16±0.02	0.17±0.01	<.0001	-0.63	-0.02 (-0.02, -0.01)	<.0001
rh_medialorbitofrontal_ThickAvg	0	197 vs 85	2.39±0.25	2.41±0.25	2.34±0.21	0.0011	0.3	0.11 (0.06, 0.16)	<.0001
rh_middletemporal_FoldInd	0	197 vs 85	94.00±48.00	84.00±40.00	121.00±44.00	<.0001	-0.88	-57.45 (-98.10, -16.80)	0.0058
rh_middletemporal_GrayVol	0	197 vs 85	11542.00±2318.00	11484.00±2386.00	11601.00±2192.00	0.2166	-0.05	105.34 (-289.99, 500.68)	0.6003
rh_middletemporal_MeanCurv	0	197 vs 85	0.15±0.02	0.14±0.01	0.16±0.01	<.0001	-2	-0.02 (-0.02, -0.02)	<.0001
rh_middletemporal_ThickAvg	0	197 vs 85	2.89±0.23	2.89±0.23	2.90±0.21	0.6376	-0.05	0.03 (-0.02, 0.07)	0.2282
rh_paracentral_FoldInd	0	197 vs 85	38.00±48.00	33.00±26.00	61.00±62.00	<.0001	-0.59	-94.96 (-190.96, 1.03)	0.0525
rh_paracentral_GrayVol	0	197 vs 85	3688.50±849.00	3691.00±889.00	3676.00±738.00	0.2932	0.02	-29.35 (-176.51, 117.81)	0.6949
rh_paracentral_MeanCurv	0	197 vs 85	0.13±0.02	0.13±0.02	0.15±0.02	<.0001	-1	-0.02 (-0.03, -0.02)	<.0001

Variable	Nmissing	Level	Total	EMBARC	APAT	P-value*	Standardized difference	Estimated difference and 95% CI	P-value**
rh_paracentral_ThickAvg	0	197 vs 85	2.37±0.22	2.34±0.22	2.43±0.21	0.0001	-0.42	-0.07 (-0.11, -0.02)	0.0021
rh_parahippocampal_FoldInd	0	197 vs 85	14.00±10.00	12.00±8.00	18.00±12.00	<.0001	-0.59	-4.84 (-24.00, 14.32)	0.6195
rh_parahippocampal_GrayVol	0	197 vs 85	2080.50±435.00	2060.00±439.00	2124.00±447.00	0.1775	-0.14	-2.37 (-86.02, 81.28)	0.9556
rh_parahippocampal_MeanCurv	0	197 vs 85	0.13±0.03	0.12±0.02	0.15±0.03	<.0001	-1.18	-0.03 (-0.03, -0.02)	<.0001
rh_parahippocampal_ThickAvg	0	197 vs 85	2.75±0.34	2.76±0.38	2.75±0.28	0.2470	0.03	-0.06 (-0.13, 0.02)	0.1393
rh_parsopercularis_FoldInd	0	197 vs 85	30.00±15.00	27.00±12.00	41.00±18.00	<.0001	-0.92	-19.39 (-97.02, 58.24)	0.6233
rh_parsopercularis_GrayVol	0	197 vs 85	3990.50±976.00	3960.00±1002.00	4056.00±800.00	0.1530	-0.11	33.88 (-142.09, 209.85)	0.7050
rh_parsopercularis_MeanCurv	0	197 vs 85	0.14±0.02	0.13±0.01	0.16±0.01	<.0001	-3	-0.02 (-0.03, -0.02)	<.0001
rh_parsopercularis_ThickAvg	0	197 vs 85	2.60±0.25	2.58±0.26	2.61±0.23	0.4764	-0.12	0.02 (-0.02, 0.06)	0.3563
rh_parsorbitalis_FoldInd	0	197 vs 85	26.00±13.00	23.00±10.00	34.00±15.00	<.0001	-0.86	-13.80 (-59.87, 32.27)	0.5559
rh_parsorbitalis_GrayVol	0	197 vs 85	2582.50±588.00	2564.00±600.00	2663.00±548.00	0.0498	-0.17	-44.76 (-139.76, 50.25)	0.3545
rh_parsorbitalis_MeanCurv	0	197 vs 85	0.17±0.03	0.16±0.02	0.19±0.02	<.0001	-1.5	-0.03 (-0.03, -0.02)	<.0001
rh_parsorbitalis_ThickAvg	0	197 vs 85	2.73±0.29	2.74±0.29	2.68±0.27	0.1139	0.21	0.07 (0.01, 0.12)	0.0173
rh_parstriangularis_FoldInd	0	197 vs 85	35.00±13.00	33.00±13.00	45.00±19.00	<.0001	-0.74	-25.59 (-40.18, -11.01)	0.0006
rh_parstriangularis_GrayVol	0	197 vs 85	4103.50±1073.00	4098.00±1132.00	4228.00±955.00	0.2539	-0.12	21.15 (-156.05, 198.35)	0.8144
rh_parstriangularis_MeanCurv	0	197 vs 85	0.15±0.02	0.14±0.01	0.16±0.01	<.0001	-2	-0.02 (-0.03, -0.02)	<.0001
rh_parstriangularis_ThickAvg	0	197 vs 85	2.48±0.21	2.48±0.23	2.49±0.17	0.8635	-0.05	0.03 (-0.01, 0.07)	0.1311
rh_pericalcarine_FoldInd	0	197 vs 85	43.00±24.00	39.00±21.00	55.00±34.00	<.0001	-0.57	-21.77 (-42.21, -1.32)	0.0370
rh_pericalcarine_GrayVol	0	197 vs 85	2165.50±668.00	2086.00±671.00	2326.00±505.00	0.0005	-0.4	-172.37 (-287.07, -57.67)	0.0034

Variable	Nmissing	Level	Total	EMBARC	APAT	P-value*	Standardized difference	Estimated difference and 95% CI	P-value**
rh_pericalcarine_MeanCurv	0	197 vs 85	0.16±0.02	0.16±0.02	0.17±0.02	<.0001	-0.5	-0.02 (-0.03, -0.02)	<.0001
rh_pericalcarine_ThickAvg	0	197 vs 85	1.58±0.25	1.53±0.22	1.68±0.22	<.0001	-0.68	-0.15 (-0.19, -0.10)	<.0001
rh_postcentral_FoldInd	0	197 vs 85	102.00±63.00	88.00±42.00	146.00±90.00	<.0001	-0.83	-115.87 (-172.84, -58.90)	<.0001
rh_postcentral_GrayVol	0	197 vs 85	8765.00±1576.00	8690.00±1604.00	8999.00±1372.00	0.0018	-0.21	-398.64 (-711.04, -86.25)	0.0126
rh_postcentral_MeanCurv	0	197 vs 85	0.13±0.02	0.13±0.01	0.15±0.01	<.0001	-2	-0.02 (-0.03, -0.02)	<.0001
rh_postcentral_ThickAvg	0	197 vs 85	2.07±0.17	2.04±0.17	2.12±0.17	<.0001	-0.47	-0.09 (-0.13, -0.06)	<.0001
rh_posteriorcingulate_FoldInd	0	197 vs 85	37.50±14.00	34.00±13.00	44.00±12.00	<.0001	-0.8	-16.69 (-35.93, 2.55)	0.0889
rh_posteriorcingulate_GrayVol	0	197 vs 85	3140.50±665.00	3094.00±733.00	3203.00±635.00	0.1128	-0.16	-38.85 (-161.25, 83.54)	0.5325
rh_posteriorcingulate_MeanCurv	0	197 vs 85	0.16±0.02	0.16±0.01	0.17±0.01	<.0001	-1	-0.02 (-0.02, -0.01)	<.0001
rh_posteriorcingulate_ThickAvg	0	197 vs 85	2.50±0.22	2.51±0.21	2.49±0.20	0.6814	0.1	0.01 (-0.03, 0.05)	0.6140
rh_precentral_FoldInd	0	197 vs 85	161.00±160.00	128.00±106.00	262.00±221.00	<.0001	-0.77	-136.83 (-245.56, -28.11)	0.0138
rh_precentral_GrayVol	0	197 vs 85	12789.50±2070.00	12722.00±2130.00	13120.00±1985.00	0.0796	-0.19	-226.27 (-603.05, 150.50)	0.2381
rh_precentral_MeanCurv	0	197 vs 85	0.13±0.03	0.13±0.02	0.16±0.01	<.0001	-1.9	-0.03 (-0.03, -0.03)	<.0001
rh_precentral_ThickAvg	0	197 vs 85	2.51±0.17	2.51±0.18	2.53±0.16	0.0434	-0.12	-0.02 (-0.05, 0.02)	0.2940
rh_precuneus_FoldInd	0	197 vs 85	101.00±46.00	93.00±44.00	117.00±39.00	<.0001	-0.58	-22.23 (-87.72, 43.26)	0.5045
rh_precuneus_GrayVol	0	197 vs 85	9613.00±1718.00	9598.00±1668.00	9853.00±1708.00	0.2235	-0.15	55.93 (-248.20, 360.06)	0.7176
rh_precuneus_MeanCurv	0	197 vs 85	0.15±0.01	0.14±0.01	0.15±0.01	<.0001	-1	-0.01 (-0.02, -0.01)	<.0001
rh_precuneus_ThickAvg	0	197 vs 85	2.39±0.19	2.37±0.18	2.42±0.16	0.0015	-0.29	-0.04 (-0.07, -0.00)	0.0311
rh_rostralanteriorcingulate_FoldInd	0	197 vs 85	24.00±10.00	22.00±9.00	28.00±13.00	<.0001	-0.54	5.17 (-20.73, 31.08)	0.6946

Variable	Nmissing	Level	Total	EMBARC	APAT	P-value*	Standardized difference	Estimated difference and 95% CI	P-value**
rh_rostralanteriorcingulate_GrayVol	0	197 vs 85	2110.50±537.00	2104.00±550.00	2138.00±565.00	0.5265	-0.06	14.70 (-96.94, 126.34)	0.7957
rh_rostralanteriorcingulate_MeanCurv	0	197 vs 85	0.16±0.02	0.16±0.02	0.17±0.02	<.0001	-0.5	-0.02 (-0.02, -0.01)	<.0001
rh_rostralanteriorcingulate_ThickAvg	0	197 vs 85	2.81±0.28	2.82±0.27	2.77±0.32	0.2512	0.17	0.05 (-0.00, 0.11)	0.0660
rh_rostralmiddlefrontal_FoldInd	0	197 vs 85	185.50±85.00	163.00±54.00	237.00±108.00	<.0001	-0.87	-182.41 (-282.83, -81.99)	0.0004
rh_rostralmiddlefrontal_GrayVol	0	197 vs 85	15321.50±2782.00	15137.00±2679.00	15409.00±2425.00	0.3429	-0.11	263.73 (-249.71, 777.18)	0.3128
rh_rostralmiddlefrontal_MeanCurv	0	197 vs 85	0.16±0.02	0.16±0.01	0.17±0.01	<.0001	-1	-0.02 (-0.02, -0.02)	<.0001
rh_rostralmiddlefrontal_ThickAvg	0	197 vs 85	2.35±0.20	2.38±0.23	2.33±0.14	0.0772	0.26	0.05 (0.01, 0.09)	0.0079
rh_superiorfrontal_FoldInd	0	197 vs 85	182.00±134.00	156.00±88.00	244.00±129.00	<.0001	-0.8	-94.76 (-452.54, 263.02)	0.6025
rh_superiorfrontal_GrayVol	0	197 vs 85	21048.50±3717.00	20893.00±3748.00	21400.00±3418.00	0.0947	-0.14	-10.68 (-618.68, 597.32)	0.9724
rh_superiorfrontal_MeanCurv	0	197 vs 85	0.15±0.02	0.14±0.01	0.16±0.01	<.0001	-2	-0.02 (-0.03, -0.02)	<.0001
rh_superiorfrontal_ThickAvg	0	197 vs 85	2.72±0.21	2.73±0.22	2.72±0.19	0.6715	0.05	0.05 (0.01, 0.08)	0.0158
rh_superiorparietal_FoldInd	0	197 vs 85	125.00±56.00	117.00±39.00	157.00±63.00	<.0001	-0.76	-74.73 (-116.36, -33.09)	0.0005
rh_superiorparietal_GrayVol	0	197 vs 85	12606.50±2283.00	12395.00±2568.00	13027.00±1699.00	0.0291	-0.29	-281.70 (-696.18, 132.79)	0.1820
rh_superiorparietal_MeanCurv	0	197 vs 85	0.14±0.01	0.14±0.01	0.15±0.01	<.0001	-1	-0.01 (-0.02, -0.01)	<.0001
rh_superiorparietal_ThickAvg	0	197 vs 85	2.18±0.17	2.15±0.18	2.23±0.20	<.0001	-0.42	-0.08 (-0.12, -0.05)	<.0001
rh_superiortemporal_FoldInd	0	197 vs 85	75.00±39.00	66.00±25.00	104.00±70.00	<.0001	-0.72	-91.30 (-126.06, -56.53)	<.0001
rh_superiortemporal_GrayVol	0	197 vs 85	11358.50±1905.00	11321.00±1990.00	11560.00±1699.00	0.0873	-0.13	-19.52 (-344.75, 305.71)	0.9060
rh_superiortemporal_MeanCurv	0	197 vs 85	0.13±0.02	0.13±0.01	0.15±0.01	<.0001	-2	-0.02 (-0.03, -0.02)	<.0001
rh_superiortemporal_ThickAvg	0	197 vs 85	2.84±0.23	2.84±0.23	2.84±0.20	0.9265	0	0.04 (-0.00, 0.08)	0.0678

Variable	Nmissing	Level	Total	EMBARC	APAT	P-value*	Standardized difference	Estimated difference and 95% CI	P-value**
rh_supramarginal_FoldInd	0	197 vs 85	86.00±29.00	81.00±27.00	97.00±31.00	<.0001	-0.55	-14.16 (-84.83, 56.52)	0.6936
rh_supramarginal_GrayVol	0	197 vs 85	9900.00±1986.00	9764.00±2051.00	10147.00±1574.00	0.0257	-0.21	-139.45 (-492.59, 213.69)	0.4376
rh_supramarginal_MeanCurv	0	197 vs 85	0.14±0.01	0.14±0.01	0.15±0.01	<.0001	-1	-0.01 (-0.02, -0.01)	<.0001
rh_supramarginal_ThickAvg	0	197 vs 85	2.59±0.20	2.57±0.18	2.62±0.17	0.0014	-0.29	-0.03 (-0.07, 0.01)	0.0932
rh_temporalpole_FoldInd	0	197 vs 85	15.00±12.00	13.00±5.00	27.00±21.00	<.0001	-0.92	-30.41 (-45.23, -15.59)	<.0001
rh_temporalpole_GrayVol	0	197 vs 85	2281.50±504.00	2335.00±509.00	2223.00±501.00	0.0174	0.22	127.88 (35.11, 220.64)	0.0071
rh_temporalpole_MeanCurv	0	197 vs 85	0.18±0.04	0.17±0.03	0.22±0.04	<.0001	-1.41	-0.05 (-0.05, -0.04)	<.0001
rh_temporalpole_ThickAvg	0	197 vs 85	3.75±0.45	3.81±0.41	3.59±0.42	<.0001	0.53	0.18 (0.09, 0.27)	<.0001
rh_transversetemporal_FoldInd	0	197 vs 85	10.00±10.00	8.00±5.00	17.00±18.00	<.0001	-0.68	-24.37 (-32.15, -16.58)	<.0001
rh_transversetemporal_GrayVol	0	197 vs 85	877.50±256.00	855.00±229.00	911.00±208.00	0.0057	-0.26	-41.39 (-83.26, 0.47)	0.0526
rh_transversetemporal_MeanCurv	0	197 vs 85	0.14±0.02	0.13±0.02	0.16±0.02	<.0001	-1.5	-0.03 (-0.03, -0.02)	<.0001
rh_transversetemporal_ThickAvg	0	197 vs 85	2.39±0.33	2.37±0.33	2.45±0.27	0.0078	-0.27	-0.05 (-0.11, 0.01)	0.0909
HDRS 17 Baseline Total	2	195 vs 85	18.00±7.00	19.00±6.00	18.00±5.00	0.1280	0.18	-	-
HDRS-17_Week8_Total	57	147 vs 78	10.00±10.00	10.00±10.00	10.00±9.00	0.7877	0	-0.60 (-2.44, 1.25)	0.5245
age	0	197 vs 85	30.00±24.00	33.00±22.00	23.66±13.12	<.0001	0.52	-	-
totedu	11	194 vs 77	15.00±3.00	15.00±3.00	15.00±2.00	0.8053	0	-0.53 (-1.19, 0.13)	0.1144
qids_eval_01	4	197 vs 81	2.00±2.00	2.00±1.00	2.00±2.00	0.2870	0	-0.18 (-0.45, 0.10)	0.2141
qids_eval_02	4	197 vs 81	2.00±2.00	2.00±2.00	2.00±2.00	0.0253	0	0.17 (-0.11, 0.46)	0.2217
qids_eval_03	4	197 vs 81	1.00±2.00	1.00±2.00	1.00±2.00	0.4777	0	-0.00 (-0.31, 0.30)	0.9752

Variable	Nmissing	Level	Total	EMBARC	APAT	P-value*	Standardized difference	Estimated difference and 95% CI	P-value**
qids_eval_04	4	197 vs 81	0.00±1.00	1.00±2.00	0.00±1.00	0.1072	0.63	0.28 (-0.10, 0.66)	0.1533
qids_eval_05	4	197 vs 81	2.00±1.00	2.00±1.00	2.00±1.00	0.0037	0	0.22 (0.07, 0.38)	0.0059
qids_eval_10	4	197 vs 81	2.00±0.00	2.00±0.00	2.00±1.00	0.0006	0	0.34 (0.17, 0.51)	0.0001
qids_eval_11	4	197 vs 81	3.00±2.00	3.00±1.00	2.00±2.00	0.0111	0.63	0.46 (0.12, 0.81)	0.0087
qids_eval_12	5	196 vs 81	1.00±1.00	1.00±2.00	1.00±1.00	0.1205	0	0.23 (0.01, 0.45)	0.0402
qids_eval_13	5	196 vs 81	2.00±2.00	2.00±1.00	1.00±1.00	<.0001	1	0.66 (0.44, 0.88)	<.0001
qids_eval_14	5	196 vs 81	2.00±0.00	2.00±0.00	2.00±1.00	<.0001	0	0.41 (0.25, 0.57)	<.0001
qids_eval_15	5	196 vs 81	1.00±2.00	1.00±1.00	1.00±1.00	<.0001	0	0.39 (0.19, 0.60)	0.0002
qids_eval_16	5	196 vs 81	1.00±1.00	1.00±2.00	1.00±1.00	0.6339	0	0.12 (-0.12, 0.36)	0.3408
qids_eval_total	5	196 vs 81	17.00±4.00	18.00±4.00	15.00±5.00	<.0001	0.66	3.26 (2.44, 4.08)	<.0001
HDRS_01	2	195 vs 85	2.00±1.00	3.00±1.00	2.00±2.00	<.0001	0.63	0.37 (0.19, 0.54)	<.0001
HDRS_23	2	195 vs 85	1.00±2.00	1.00±2.00	2.00±2.00	<.0001	-0.5	-1.36 (-1.61, -1.11)	<.0001
HDRS_07	2	195 vs 85	1.00±1.00	1.00±2.00	2.00±1.00	<.0001	-0.63	-1.31 (-1.49, -1.12)	<.0001
HDRS_22	2	195 vs 85	2.00±1.00	2.00±1.00	2.00±1.00	0.3083	0	0.07 (-0.12, 0.26)	0.4855
HDRS_04	2	195 vs 85	2.00±1.00	2.00±1.00	2.00±2.00	<.0001	0	0.54 (0.34, 0.74)	<.0001
HDRS_05	2	195 vs 85	2.00±2.00	2.00±1.00	1.00±2.00	<.0001	0.63	1.31 (1.07, 1.54)	<.0001
HDRS_06	2	195 vs 85	1.00±2.00	1.00±2.00	0.00±2.00	0.0002	0.5	0.36 (0.14, 0.58)	0.0013
HDRS_12	2	195 vs 85	0.00±1.00	0.00±1.00	0.00±1.00	0.0615	0	-0.14 (-0.37, 0.09)	0.2345
HDRS_16	2	195 vs 85	0.00±0.00	0.00±1.00	0.00±0.00	0.0026	0	0.20 (0.03, 0.36)	0.0202

Variable	Nmissing	Level	Total	EMBARC	APAT	P-value*	Standardized difference	Estimated difference and 95% CI	P-value**
HDRS_13	2	195 vs 85	1.00±2.00	1.00±2.00	2.00±1.00	<.0001	-0.63	-0.67 (-0.88, -0.45)	<.0001
HDRS_14	2	195 vs 85	0.00±1.00	0.00±1.00	0.00±2.00	0.0003	0	-0.40 (-0.59, -0.21)	<.0001
HDRS_02	2	195 vs 85	2.00±1.00	2.00±1.00	2.00±1.00	<.0001	0	0.68 (0.49, 0.87)	<.0001
HDRS_24	2	195 vs 85	2.00±1.00	2.00±1.00	2.00±1.00	0.5128	0	0.11 (-0.11, 0.32)	0.3366
HDRS_03	2	195 vs 85	2.00±1.00	3.00±1.00	1.00±2.00	<.0001	1.26	1.55 (1.37, 1.73)	<.0001
HDRS_10	2	195 vs 85	1.00±2.00	1.00±1.00	2.00±2.00	<.0001	-0.63	-1.37 (-1.57, -1.17)	<.0001
HDRS_11	2	195 vs 85	0.00±1.00	0.00±1.00	1.00±1.00	<.0001	-1	-0.96 (-1.15, -0.78)	<.0001
HDRS_15	53	144 vs 85	0.00±0.00	0.00±0.00	0.00±1.00	0.0001	0	-0.37 (-0.56, -0.19)	<.0001
HDRS_09	2	195 vs 85	0.00±1.00	0.00±0.00	1.00±1.00	<.0001	-1.41	-0.43 (-0.63, -0.23)	<.0001
HDRS_08	2	195 vs 85	0.00±1.00	1.00±2.00	0.00±0.00	<.0001	0.71	0.51 (0.32, 0.71)	<.0001
HDRS_19	2	195 vs 85	2.00±3.00	3.00±2.00	0.00±0.00	<.0001	2.12	2.54 (2.30, 2.78)	<.0001
HDRS_20	2	195 vs 85	0.00±2.00	1.00±2.00	0.00±0.00	<.0001	0.71	0.88 (0.69, 1.07)	<.0001
HDRS_21	2	195 vs 85	1.00±2.00	2.00±1.00	0.00±0.00	<.0001	2.83	1.54 (1.37, 1.72)	<.0001
HDRS_F1_AS	2	195 vs 85	12.00±4.00	13.00±4.00	11.00±5.00	<.0001	0.44	1.93 (1.27, 2.59)	<.0001
HDRS_F2_AS	2	195 vs 85	3.00±3.00	2.00±2.00	4.00±3.00	<.0001	-0.78	-1.64 (-1.99, -1.30)	<.0001
HDRS_F3_AS	57	140 vs 85	4.00±7.00	6.00±2.00	0.00±0.00	<.0001	4.24	5.26 (4.86, 5.66)	<.0001
HDRS_F4_AS	53	144 vs 85	2.00±3.00	1.00±2.00	5.00±2.00	<.0001	-2	-3.17 (-3.56, -2.79)	<.0001
HDRS_F5_AS	2	195 vs 85	5.00±3.00	5.00±2.00	3.00±2.00	<.0001	1	2.21 (1.84, 2.57)	<.0001
HDRS_F1_LWS	2	195 vs 85	7.09±2.49	7.29±2.40	6.30±2.97	<.0001	0.37	1.02 (0.63, 1.42)	<.0001

Variable	Nmissing	Level	Total	EMBARC	APAT	P-value*	Standardized difference	Estimated difference and 95% CI	P-value**
HDRS_F2_LWS	2	195 vs 85	1.34±1.42	1.16±1.42	2.10±1.34	<.0001	-0.68	-0.72 (-0.97, -0.47)	<.0001
HDRS_F3_LWS	57	140 vs 85	2.18±3.68	3.27±1.69	0.00±0.00	<.0001	2.74	2.88 (2.64, 3.11)	<.0001
HDRS_F4_LWS	53	144 vs 85	1.48±1.98	0.74±1.46	2.90±1.58	<.0001	-1.42	-1.94 (-2.20, -1.69)	<.0001
HDRS_F5_LWS	2	195 vs 85	3.49±2.10	3.97±1.66	2.07±1.96	<.0001	1.05	1.70 (1.43, 1.97)	<.0001
HDRS_18	2	195 vs 85	2.00±1.00	2.00±1.00	2.00±3.00	0.0067	0	-0.46 (-0.74, -0.18)	0.0014
HDRS_17	57	140 vs 85	0.00±0.00	0.00±0.00	0.00±0.00	<.0001	.	0.24 (0.11, 0.38)	0.0005
Placebo	0	0	136 (48.23%)	94 (47.72%)	42 (49.41%)	0.7937	0.03	-	-
		1	146 (51.77%)	103 (52.28%)	43 (50.59%)			-	

*: For categorical variables, p-values were based on Chi-squared test with exact p-value from Monte Carlo simulation; for continuous variable, p-value was based on Wilcoxon rank sum test.
**: P-value was based on multiple linear regression adjusted for age, sex, and Baseline HDRS17.
Note: For continuous variable, median+/-IQR were reported.

A4: Example R code for calculating final p-value for comparing different ROC curves and combining ROC curves from imputed datasets

```
### Step 1: ROC curve for single dataset
my_ROC = function(data, y_predict, y_true, pos_class, neg_class, thresh){
  thresholds = thresh
  n_neg = length(y_true[which(y_true == neg_class)])
  n_pos = length(y_true[which(y_true == pos_class)])
  res = NULL
  for (threshold in thresholds){
    tp = length(y_predict[which(y_predict >= threshold & y_true == pos_class)])
    fp = length(y_predict[which(y_predict >= threshold & y_true == neg_class)])
    tn = length(y_predict[which(y_predict < threshold & y_true == neg_class)])
    fn = length(y_predict[which(y_predict < threshold & y_true == pos_class)])
    tpr = tp/n_pos
    fpr = fp/n_neg
    curr = c(threshold, tpr, fpr)
    res = rbind(res, curr)
  }
  res = data.frame(res)
  colnames(res) = c("threshold", "tpr", "fpr")
  resauc = auc(y_true, y_predict, direction="<")
  return(list(res=res, resauc=resauc))
```

```
}

### Step 2: ROC curve for multiple datasets, calculate mean_tpr and mean_fpr group by thresholds
# check invalid points
invalid_points = function(res){
  invalid_tpr = 0
  invalid_fpr = 0
  for (i in 2:nrow(res)){
    if (res$mean_tpr[i-1] < res$mean_tpr[i]){
      invalid_tpr = invalid_tpr + 1
    }
    if (res$mean_fpr[i-1] < res$mean_fpr[i]){
      invalid_fpr = invalid_fpr + 1
    }
  }
  return(list(invalid_tpr=invalid_tpr, invalid_fpr=invalid_fpr))
}

# function
merge_ROC_res = function(indat, name, threshold_i){
  dats = unique(indat[, name])
  comb_res = NULL
  comb_auc = NULL
  for (i in dats){
    curr_dat = indat[which(indat[, name] == i),]
    curr1 = my_ROC(data = curr_dat, y_predict = curr_dat$y_predict, y_true = curr_dat$y_true, pos_class
= 1, neg_class = 0, thresh = threshold_i)
    curr_res = curr1$res
    curr_auc = curr1$resauc
    curr_res$datname = i
    comb_res = rbind(comb_res, curr_res)
    comb_auc = c(comb_auc, curr_auc)
  }
  out_res = comb_res %>% group_by(threshold) %>% summarise(mean_tpr = round(mean(tpr), 2),
mean_fpr = round(mean(fpr), 2)) %>% as.data.frame
  mean_auc = mean(comb_auc)
  inv = invalid_points(out_res)
  if (inv$invalid_tpr + inv$invalid_fpr > 0){
    print("Not valid plot, change the thresholds_interval.")
  }
  return(list(out_res=out_res, out_auc=mean_auc))
}

### Step 3: consider categories such as gender, medication history, site, etc.
ROC_classes = function(indata, group_name, threshold_interval){
  groups = unique(indata[, group_name])
  resout = NULL
  resauc = NULL
  for (g in groups){
    subdat = indata[which(indata[, group_name] == g),]
    sub = merge_ROC_res(indat = subdat, name = "X_Imputation_", threshold_i = threshold_interval)
```

```
    subres = sub$out_res
    subres[, group_name] = g
    resout = rbind(resout, subres)

    subauc = c(g, sub$out_auc)
    resauc = rbind(resauc, subauc)
  }
  return(list(resout=resout, resauc=resauc))
}

### step 4: calculate combined p-values based on Fisher's method
pvalues = function(g){
  out = rep(NA, 10)
  for (i in 1:10){
    inputi = input[which(input$X_Imputation_ == i),]
    out[i] = compareROCindep(inputi$y_predict, inputi[, g], inputi$y_true)$p.value
  }
  out_F = round(hmp.stat(out), 4)
  print(out)
  return(out_F)
}

### Example of ROC curves by sex among 1/3 APAT+EMBARC testing data
res_gender = ROC_classes(indat = input, group_name = "sex", threshold_interval = cutoffs)
res_gender1 = res_gender$resout
n1 = input %>% count(sex)

auc1 = data.frame(res_gender[["resauc"]])
colnames(auc1) = c("sex", "auc")
label1 = merge(auc1, n1, by = "sex")

res_gender1$sex = ifelse(res_gender1$sex == "Female", paste0("Female", ", AUC=",
round(as.numeric(label1[which(label1$sex == "Female"),2]), 4), " (N=", label1[which(label1$sex ==
"Female"),3]/10, ")"), paste0("Male", ", AUC = ", round(as.numeric(label1[which(label1$sex ==
"Male"),2]), 4), " (N=", label1[which(label1$sex == "Male"),3]/10, ")"))

pval1 = pvalues("sex")

p1 = ggplot(res_gender1, aes(x = `mean_fpr`, y = `mean_tpr`, color = `sex`)) +
  geom_path(aes(color = `sex`)) +
  ggtitle("A") +
  geom_abline (intercept = 0, slope = 1) +
  scale_x_continuous("1-Specificity") +
  scale_y_continuous("Sensitivity") +
  theme(legend.position = c(0.75, 0.15),
        legend.text=element_text(size=10),
        legend.background = element_blank(),
        legend.box.background = element_blank(),
        legend.key = element_blank()) +
  annotate("text", x = .2, y = .75, label = paste0("p-value = ", pval1), size=10.5/.pt) + labs(color = "Sex")
```

A5: Example Python code for applying our predictive modeling pipeline

```
### Step 1. Define set of hyperparameters to be selected for cross validation
base_models = {'Random Forest': {'Model': RandomForestClassifier(), 'cv_parm': {'n_estimators': [1000],
'max_features': ['auto', 'sqrt', 'log2'], 'max_depth': [2,3,4,5], 'criterion': ['gini'], 'random_state': [42]}},
   'Gradient Boosting': {'Model': GradientBoostingClassifier(), 'cv_parm': {"loss": ["deviance"],
"learning_rate": [0.01, 0.05, 0.1, 0.2], "max_depth": [2,3,4,5], "max_features":["log2","sqrt"], "criterion":
["friedman_mse",  "mse"], "subsample": [1.0], "n_estimators": [1000], 'random_state': [42]}},
   'XGBoost': {'Model': xgb.XGBClassifier(), 'cv_parm': {'objective':['reg:logistic'], 'learning_rate': [0.1,
0.3, 1], 'max_depth': [2,3,4,5], 'subsample': [1], 'colsample_bytree': [1], 'n_estimators': [1000],
'random_state': [42]}},
   'SVM': {'Model': svm.SVC(), 'cv_parm': {'kernel': ['sigmoid'], 'probability': [True], 'random_state':
[42]}},
   'Penalized Logistic': {'Model': LogisticRegression(), 'cv_parm': {'penalty': ['l1','l2'], 'C': [0.001, 0.01,
0.1, 1, 10], 'solver': ['liblinear'], 'random_state': [42]}},
   'Neural Network': {'Model': MLPClassifier(), 'cv_parm': {'solver': ['lbfgs'], 'max_iter': [500],
'hidden_layer_sizes': [(100,50)], 'activation': ['relu'], 'random_state': [42]}}}

### Step 2. Standardization function for training and testing data
def scale_X(X_train, X_test):
   scaler = StandardScaler()
   X_train_scaled, X_test_scaled = X_train.copy(), X_test.copy()
   X_train_scaled = scaler.fit_transform(X_train_scaled.values)
   X_test_scaled = scaler.transform(X_test_scaled.values)
   return X_train_scaled, X_test_scaled

### Step 3. Modeling to obtain performances for training data
rkf = RepeatedKFold(n_splits=4, n_repeats=5, random_state=123)

res_all = {}
for i in range(1,11):
   outdat = BMs[BMs["_Imputation_"] == i]
   rem = y
   pre = X[var] # only keep top 50 predictors
   print("dataset {}".format(i))

   res = {'Random Forest': {'AUC': [], 'Metrics': [], 'Importance Ranking': pd.DataFrame()},
        'Gradient Boosting': {'AUC': [], 'Metrics': [], 'Importance Ranking': pd.DataFrame()},
        'SVM': {'AUC': [], 'Metrics': [], 'Importance Ranking': pd.DataFrame()},
        'XGBoost': {'AUC': [], 'Metrics': [], 'Importance Ranking': pd.DataFrame()},
        'Penalized Logistic': {'AUC': [], 'Metrics': [], 'Importance Ranking': pd.DataFrame()},
        'Neural Network': {'AUC': [], 'Metrics': [], 'Importance Ranking': pd.DataFrame()},
        'Stacking': {'AUC': [], 'Metrics': []}}

   for train, test in rkf.split(outdat):
      train_pre, test_pre = pre.iloc[train, :], pre.iloc[test, :]
      train_rem, test_rem = rem[train], rem[test]
      train_pre_scaled, test_pre_scaled = scale_X(train_pre, test_pre)

      pred_train, pred_test = pd.DataFrame(), pd.DataFrame()
      pred_train_2, pred_test_2 = pd.DataFrame(), pd.DataFrame()
```

```
        for name, model_cv in base_models.items():
            grid_search = GridSearchCV(estimator = model_cv['Model'], param_grid = model_cv['cv_parm'], 
scoring = 'roc_auc', cv = 3)
            grid_search.fit(train_pre_scaled, train_rem)
            prob_xtrain = grid_search.predict_proba(train_pre_scaled)
            prob_xtest = grid_search.predict_proba(test_pre_scaled)
            pred_train = pd.concat([pred_train, pd.DataFrame(prob_xtrain)[1]], axis=1).fillna(np.nan)
            pred_test = pd.concat([pred_test, pd.DataFrame(prob_xtest)[1]], axis=1).fillna(np.nan)

            if name == "Ridge" or name == "Neural Network":
                pred_train_2 = pd.concat([pred_train_2, pd.DataFrame(prob_xtrain)[1]], axis=1).fillna(np.nan)
                pred_test_2 = pd.concat([pred_test_2, pd.DataFrame(prob_xtest)[1]], axis=1).fillna(np.nan)

            prediction = grid_search.predict(test_pre_scaled)
            auc = float(format(roc_auc_score(test_rem, prob_xtest[:,1]), '.4f'))
            cnfm = confusion_matrix(test_rem, prediction)

            res[name]['AUC'].append((auc))
            res[name]['Metrics'].append((cnfm.flatten()))

        stack = LogisticRegression(penalty='none', random_state = 42)
        stack.fit(pred_train, train_rem)
        prediction = stack.predict(pred_test)
        auc = float(format(roc_auc_score(test_rem, stack.predict_proba(pred_test)[:,1]), '.4f'))
        cnfm = confusion_matrix(test_rem, prediction)
        res['Stacking']['AUC'].append(auc)
        res['Stacking']['Metrics'].append(cnfm.flatten())

        stack2 = LogisticRegression(penalty='none', random_state = 42)
        stack2.fit(pred_train_2, train_rem)
        prediction2 = stack2.predict(pred_test_2)
        auc_2 = float(format(roc_auc_score(test_rem, stack2.predict_proba(pred_test_2)[:,1]), '.4f'))
        cnfm_2 = confusion_matrix(test_rem, prediction2)
        res['Stacking_2']['AUC'].append(auc_2)
        res['Stacking_2']['Metrics'].append(cnfm_2.flatten())
    res_all[i] = res

### Step 4. Modeling to obtain performances for testing data
def bootstrap_auc_ci(y_true, y_scores, n_bootstrap_samples=1000, random_seed=None):
    np.random.seed(random_seed)
    aucs = []
    for _ in range(n_bootstrap_samples):
        indices = np.random.choice(len(y_true), len(y_true), replace=True)
        y_true_bootstrap = np.array(y_true)[indices]
        y_scores_bootstrap = np.array(y_scores)[indices]
        auc_bootstrap = roc_auc_score(y_true_bootstrap, y_scores_bootstrap)
        aucs.append(auc_bootstrap)
    auc_se = np.std(aucs) / np.sqrt(len(aucs))
    return auc_se
```

```
res = {'Random Forest': {'AUC': [], 'AUC_se': [], 'Metrics': [], 'Importance Ranking': pd.DataFrame()},
'Gradient Boosting': {'AUC': [], 'AUC_se': [], 'Metrics': [], 'Importance Ranking': pd.DataFrame()},
'SVM': {'AUC': [], 'AUC_se': [], 'Metrics': [], 'Importance Ranking': pd.DataFrame()}, 'XGBoost':
{'AUC': [], 'AUC_se': [], 'Metrics': [], 'Importance Ranking': pd.DataFrame()}, 'Penalized Logistic':
{'AUC': [], 'AUC_se': [], 'Metrics': [], 'Importance Ranking': pd.DataFrame()}, 'Neural Network': {'AUC':
[], 'AUC_se': [], 'Metrics': [], 'Importance Ranking': pd.DataFrame()}, 'Stacking': {'AUC': [], 'AUC_se': [],
'Metrics': []}, 'Stacking_2': {'AUC': [], 'AUC_se': [], 'Metrics': []}}

for i in range(1,11):
    outdat, EMBARC1 = BMs[BMs["_Imputation_"] == i], EMBARC[EMBARC["_Imputation_"] == i]
    rem, pre = train_y, train_X[var] # only keep top 50 predictors
    embarc_y, embarc_X = test_y, test_X[var]

    train_pre, train_rem = pre, rem
    train_pre_scaled, embarc_X_scale = scale_X(train_pre, embarc_X)

    pred_train, pred_test = pd.DataFrame(), pd.DataFrame()
    pred_train_2, pred_test_2 = pd.DataFrame(), pd.DataFrame()
    for name, model_cv in base_models.items():
        grid_search = GridSearchCV(estimator = model_cv['Model'], param_grid = model_cv['cv_parm'],
scoring = 'roc_auc', cv = 4)
        grid_search.fit(train_pre_scaled, train_rem)
        prob_xtrain = grid_search.predict_proba(train_pre_scaled)
        prob_xtest = grid_search.predict_proba(embarc_X_scale)
        pred_train = pd.concat([pred_train, pd.DataFrame(prob_xtrain)[1]], axis=1).fillna(np.nan)
        pred_test = pd.concat([pred_test, pd.DataFrame(prob_xtest)[1]], axis=1).fillna(np.nan)
        if name == "Ridge" or name == "Neural Network":
            pred_train_2 = pd.concat([pred_train_2, pd.DataFrame(prob_xtrain)[1]], axis=1).fillna(np.nan)
            pred_test_2 = pd.concat([pred_test_2, pd.DataFrame(prob_xtest)[1]], axis=1).fillna(np.nan)
        prediction = grid_search.predict(embarc_X_scale)
        auc = float(format(roc_auc_score(embarc_y, prob_xtest[:,1]), '.4f'))
        auc_se = bootstrap_auc_ci(embarc_y, prob_xtest[:,1])
        cnfm = confusion_matrix(embarc_y, prediction)
        res[name]['AUC'].append((auc))
        res[name]['AUC_se'].append((auc_se))
        res[name]['Metrics'].append((cnfm.flatten()))

    stack = LogisticRegression(penalty='none', random_state = 42)
    stack.fit(pred_train, train_rem)
    prediction = stack.predict(pred_test)
    auc = float(format(roc_auc_score(embarc_y, stack.predict_proba(pred_test)[:,1]), '.4f'))
    auc_se = bootstrap_auc_ci(embarc_y, stack.predict_proba(pred_test)[:,1])
    cnfm = confusion_matrix(embarc_y, prediction)
    res['Stacking']['AUC'].append(auc)
    res[name]['AUC_se'].append((auc_se))
    res['Stacking']['Metrics'].append(cnfm.flatten())

    stack2 = LogisticRegression(penalty='none', random_state = 42)
    stack2.fit(pred_train_2, train_rem)
    prediction2 = stack2.predict(pred_test_2)
    auc_2 = float(format(roc_auc_score(embarc_y, stack2.predict_proba(pred_test_2)[:,1]), '.4f'))
```

```
    auc_se_2 = bootstrap_auc_ci(embarc_y, stack3.predict_proba(pred_test_2)[:,1])
    cnfm_2 = confusion_matrix(embarc_y, prediction2)
    res['Stacking_2']['AUC'].append(auc_2)
    res['Stacking_2']['AUC_se'].append(auc_se_2)
    res['Stacking_2']['Metrics'].append(cnfm_2.flatten())

### Step 4. Rubin's rule for training data
def RubinRule(dataname, methodname, performname):
    sub_dat = dataname.loc[dataname['Method'] == methodname]
    Q = [0]*10
    U = [0]*10
    for i in range(10):
        sub_dat_i = sub_dat.iloc[i*5:i*5+5,:]
        Q[i] = np.mean(sub_dat_i[performname])
        U[i] = (sem(sub_dat_i[performname]))**2
    Qbar = np.mean(Q)
    Ubar = np.mean(U)
    B = variance(Q)
    T = Ubar + (1+1/10)*B
    out_mod = str(float(format(Qbar, '.4f'))) + ' (' + str(float(format(np.sqrt(T), '.4f'))) + ')'
    return out_mod

def AUC_rubin(dataname, method_name):
    sub_dat = dataname.loc[dataname['Method'] == method_name]
    Q = [0]*10
    U = [0]*10
    for i in range(10):
        sub_dat_i = sub_dat.iloc[i*20:i*20+20,:]
        Q[i] = np.mean(sub_dat_i['AUC'])
        U[i] = (sem(sub_dat_i['AUC']))**2
    Qbar = np.mean(Q)
    Ubar = np.mean(U)
    B = variance(Q)
    T = Ubar + (1+1/10)*B
    auc_mod = str(float(format(Qbar, '.4f'))) + ' (' + str(float(format(np.sqrt(T), '.4f'))) + ')'
    return auc_mod

### Step 5. Rubin's rule for testing data
def Rubin_performance(performance, n):
    performance = performance.reset_index(drop=True)
    n = n.reset_index(drop=True)
    Q = [0]*10
    U = [0]*10
    for i in range(10):
        Q[i] = performance[i]
        U[i] = performance[i] * (1-performance[i]) / n[i]
    Qbar = np.mean(Q)
    Ubar = np.mean(U)
    B = variance(Q)
    T = Ubar + (1+1/10)*B
    out_mod = str(float(format(Qbar, '.4f'))) + ' (' + str(float(format(np.sqrt(T), '.4f'))) + ')'
```

```
    return out_mod

def AUC_mod(data, method):
    subdat = data.loc[data['Method'] == method]
    performance = subdat['AUC'].reset_index(drop=True)
    se = subdat['AUC_se'].reset_index(drop=True)
    Q = [0]*10
    U = [0]*10
    for i in range(10):
        Q[i] = performance[i]
        U[i] = se[i]**2
    Qbar = np.mean(Q)
    Ubar = np.mean(U)
    B = variance(Q)
    T = Ubar + (1+1/10)*B
    out_mod = str(float(format(Qbar, '.4f'))) + ' (' + str(float(format(np.sqrt(T), '.4f'))) + ')'
    return out_mod
```
